# Macrophage–endothelial cell crosstalk orchestrates neutrophil recruitment in inflamed mucosa

**DOI:** 10.1172/JCI170733

**Published:** 2023-08-01

**Authors:** Xingsheng Ren, Laura D. Manzanares, Enzo B. Piccolo, Jessica M. Urbanczyk, David P. Sullivan, Lenore K. Yalom, Triet M. Bui, Connor Lantz, Hinda Najem, Parambir S. Dulai, Amy B. Heimberger, Edward B. Thorp, Ronen Sumagin

**Affiliations:** 1 Department of Pathology, Northwestern University Feinberg School of Medicine, Chicago, Illinois, USA.; 2 Department of Neurological Surgery and Malnati Brain Tumor Institute of the Lurie Comprehensive Cancer Center, Feinberg School of Medicine, Northwestern University, Chicago, Illinois, USA.; 3 Department of Medicine, Gastroenterology and Hepatology, Northwestern Memorial Hospital, Chicago, Illinois, USA.

**Keywords:** Cell Biology, Inflammation, Cell migration/adhesion, Macrophages, Neutrophils

## Abstract

Neutrophil (PMN) mobilization to sites of insult is critical for host defense and requires transendothelial migration (TEM). TEM involves several well-studied sequential adhesive interactions with vascular endothelial cells (ECs); however, what initiates or terminates this process is not well-understood. Here, we describe what we believe to be a new mechanism where vessel-associated macrophages through localized interactions primed EC responses to form ICAM-1 “hot spots” to support PMN TEM. Using real-time intravital microscopy of LPS-inflamed intestines in CX3CR1-EGFP macrophage-reporter mice, complemented by whole-mount tissue imaging and flow cytometry, we found that macrophage vessel association is critical for the initiation of PMN-EC adhesive interactions, PMN TEM, and subsequent accumulation in the intestinal mucosa. Anti–colony stimulating factor 1 receptor Ab-mediated macrophage depletion in the lamina propria and at the vessel wall resulted in elimination of ICAM-1 hot spots impeding PMN-EC interactions and TEM. Mechanistically, the use of human clinical specimens, TNF-α–KO macrophage chimeras, TNF-α/TNF receptor (TNF-α/TNFR) neutralization, and multicellular macrophage-EC-PMN cocultures revealed that macrophage-derived TNF-α and EC TNFR2 axis mediated this regulatory mechanism and was required for PMN TEM. As such, our findings identified clinically relevant mechanisms by which macrophages regulate PMN trafficking in inflamed mucosa.

## Graphical abstract

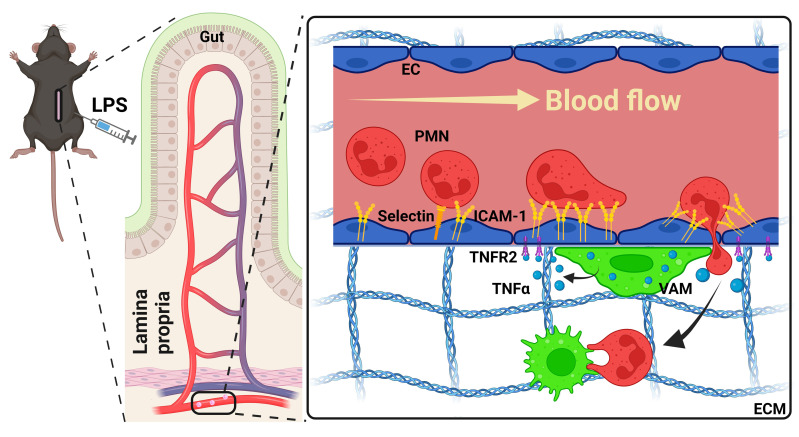

## Introduction

Mounting an efficient immune response is critical for host defense; however, dysregulated immune cell recruitment and the resulting maladaptive inflammation are hallmarks of many pathological conditions. Neutrophils (PMNs) are first responder effector cells specialized in dealing with infectious challenges, but their accumulation and activity in tissues can result in exacerbated inflammation and tissue damage. Such is the case reported for COVID-19 infections, sepsis, and inflammatory bowel diseases (IBD), in which PMN numbers have been correlated with disease severity (1–3).

PMNs implement their effector functions once they exit the circulation and enter the underlying tissue. Thus, crossing the endothelial cell (EC) barrier is the first critical regulatory step for PMN recruitment and function. PMN transendothelial migration (TEM) requires several sequential adhesive steps initiated with selectin-mediated rolling, followed by ICAM-1/VCAM-mediated adhesion and luminal crawling, and terminating with PECAM-1–dependent EC crossing (4–6). Although key players and signaling pathways meditating PMN-EC interactions during the PMN recruitment cascade have been extensively studied, mechanisms involved in the initiation and termination of this process are less understood.

Macrophages in the brain have been previously shown to help regulate vascular permeability and maintain the blood-brain barrier (7–10). Moreover, in skin infections, vessel-associated macrophages (VAMs) have been shown to release chemokines to direct PMN trafficking (11). Thus, VAMs may serve as important novel regulators of vascular function, providing specific cues for the initiation of the PMN recruitment cascade.

Gut mucosa is densely populated with resident macrophages that fulfill unique protective functions, including combating pathogens by phagocytosis and presenting antigens to activate the adaptive immune response as well as interact with the enteric nervous system to regulate gut motility and secretion (12–14). Both tissue-resident and recruited inflammatory macrophages are primarily derived from blood monocytes, with the exception of a self-renewing Trim4^+^CD4^+^ cell subset (15, 16). Macrophages are incredibly plastic and can promote the initiation and resolution of inflammation via the release of inflammatory cytokines, including various ILs and TNF-α, or prorepair factors, such as TGF-β, IL-10, and resolvins (17–24). As with the brain, gut macrophages have also been found to associate with blood vessels. During homeostasis, macrophage localization to blood vessels was found to be dependent on nuclear receptor subfamily 4 group A member 1 (NR4A1) signaling and regulated by the microbiome (25). However, the underlying mechanisms of VAM-EC communication are still not defined.

We found that LPS stimulation, simulating endotoxic insult as seen in sepsis or microbial dysbiosis in IBD, resulted in a robust increase in the number of VAMs in inflamed intestines. Our findings further implicated VAMs in a unique crosstalk with inflamed ECs to promote PMN recruitment in inflamed mucosa. Specifically, VAMs were found to promote highly localized induction in EC ICAM-1 expression via the TNF-α/TNFR2 axis, creating “hot spots” for the initiation of EC-PMN adhesive interactions. ICAM-1, which is basally expressed at low levels, has been shown to be significantly upregulated in inflamed ECs (26, 27). However, our studies link VAMs and specific, localized priming of vascular ECs to the preferential accommodation of PMN TEM and recruitment into inflamed mucosa.

## Results

### Macrophages promote PMN adhesion and TEM in inflamed intestinal mucosa.

**Figure 1:**
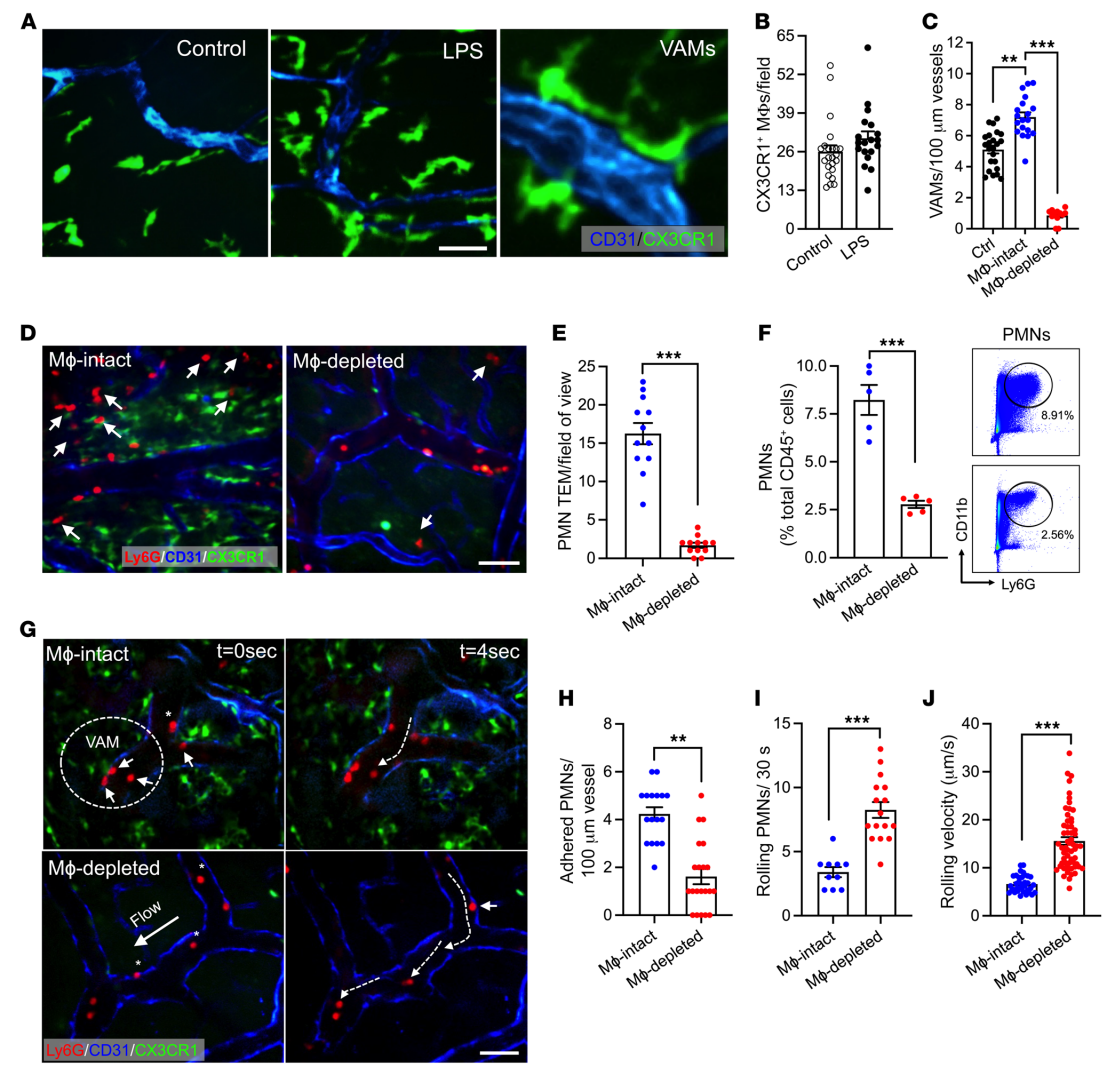
Macrophages promote PMN adhesion and TEM in inflamed intestinal mucosa. Inflammation of the intestinal mucosa was induced by i.p. administration of LPS (100 μg, 24 hours). (**A**) Representative whole-mount confocal microscopy images using CX3CR-EGFP reporter mice showing interstitial macrophage recruitment and association with blood vessels during LPS-induced inflammation. Scale bar: 20 μm. (**B**) Quantification of CX3CR1^+^ macrophage (MΦ) numbers per field of view and (**C**) VAMs per vessel length. Blood vessels were visualized using PECAM-1 (CD31, blue) staining. (**D**–**J**) Intravital microscopy (IVM) of inflamed intestines was performed on CX3CR1-EGFP mice with and without macrophage depletion with a CSF-1R Ab (400 μg/mouse, every other day for 3 weeks). PMNs were labeled by a low dose of fluorescently labeled anti-Ly6G Ab (2 μg, i.v.). (**D**) Representative images of VAM depletion and PMN tissue infiltration by IVM. Scale bar: 20 µm. Arrows indicate extravasated PMNs. (**E**) Quantification of tissue PMNs by IVM and (**F**) by flow cytometry of digested intestinal lamina propria. A representative flow diagram is shown (right). (**G**) Representative time-lapse images (based on a real-time acquisition) show decreased adherent PMN and increased displacement (dotted white arrows) of rolling PMNs in macrophage-depleted animals. Solid white arrows denote adherent PMNs in area of VAM-EC contact. Scale bar: 20 μm. (**H**) Quantification of adherent and (**I**) rolling PMN (per 30 seconds) and (**J**) rolling velocities of individual PMNs with and without macrophage depletion. For whole-mount preparation, images are representative of *n* = 4–5 mice per condition. For IVM, *n* = 3–5 mice per condition. **P < 0.01, ***P < 0.001. Two-sided Student’s *t* test. Data represent mean ± SEM.

Macrophages densely populate the intestinal mucosa and play important roles in maintaining tissue homeostasis in health and disease (13, 28–30). To visualize and establish the spatial localization of interstitial macrophages in LPS-inflamed intestines, we performed whole-mount confocal microscopy imaging in CX3CR1-EGFP macrophage reporter mice. Interestingly, we found that while the total number of interstitial macrophages was not significantly changed with LPS stimulation, the number of VAMs was significantly increased relative to untreated tissue (5.1 ± 1.4 to 7.2 ± 1.7, respectively, Figure 1, A–C). VAMs were defined as all CX3CR1-EGFP macrophages in direct contact with the vessel wall per visualization by microscopy. PMN colabeling in these mice (low dose of fluorescently labeled anti-Ly6G Ab, 2 μg, i.v.) revealed frequent attachment/accumulation of circulating PMNs at the vascular regions that were in contact with VAMs (representative images, Figure 1G, dotted circle; 75% ± 2.9% of vascular regions with 2 or more attached PMNs were regions of VAM-EC contact, Supplemental Figure 1A; supplemental material available online with this article; https://doi.org/10.1172/JCI170733DS1). To address the hypothesis that in inflamed intestinal mucosa VAMs may facilitate PMN-EC interactions and PMN TEM, PMN infiltration of the intestinal mucosa was quantified with and without CSF-1R Ab–mediated depletion of intestinal interstitial macrophages. Efficient macrophage depletion in the intestinal tissue and specifically that of VAMs (>90%, 400 μg Ab, i.p., every other day for 3 weeks) was confirmed by whole-mount imaging (Figure 1, C and D) and flow cytometry (Supplemental Figure 1B). Macrophage depletion significantly reduced the number of tissue-infiltrating PMNs (~4-fold, Figure 1, D and E) following LPS stimulation, supporting the role of macrophages in regulating PMN TEM. Reduction in PMN numbers in inflamed intestinal mucosa was also confirmed by flow cytometry, gating on CD45^+^Ly6G^+^CD11b^+^ PMNs (Figure 1F and Supplemental Figure 1C). Consistent with enhanced granulopoiesis in response to systemic inflammation and an impairment in PMN TEM, flow cytometry revealed elevated numbers of PMNs in the circulation and in the BM in LPS-stimulated macrophage-depleted mice (Supplemental Figure 1, D and E).

We next used intravital microscopy of the intestine to visualize and assess in real-time whether VAMs promote PMN adhesive interactions with inflamed ECs. In the absence of macrophages, PMN adhesion to vascular ECs was significantly reduced (Figure 1, G and H, and Supplemental Videos 1 and 2). Consistent with impaired attachment, the number of rolling PMNs was significantly increased and rolling velocities were significantly elevated (Figure 1, I and J), suggesting lesser PMN and/or EC activation. These observations show that VAMs promote PMN adhesion and TEM in inflamed intestinal mucosa.

### VAMs prime gut EC activation.

**Figure 2:**
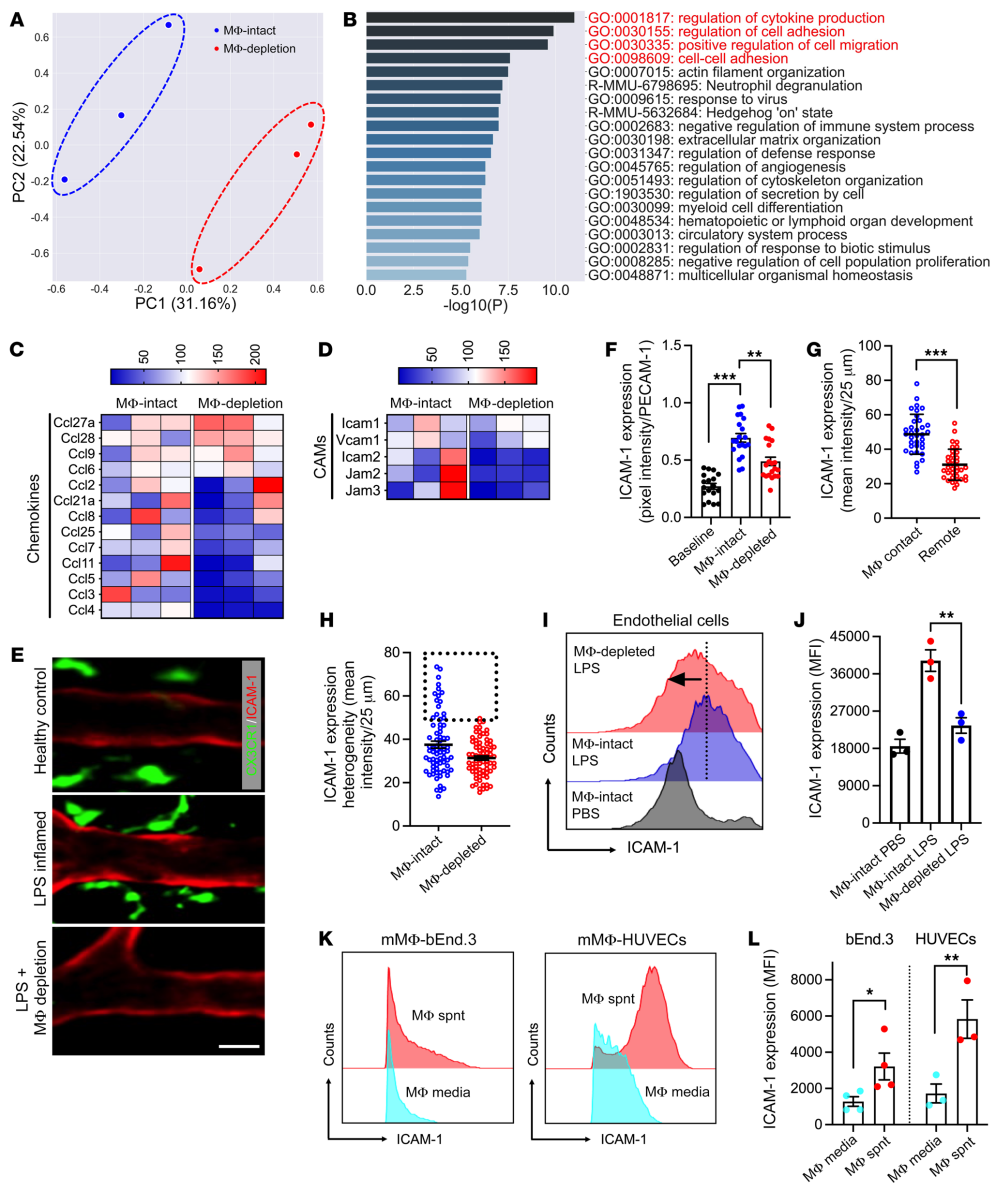
VAMs prime gut EC activation. (**A**–**D**) CD45^–^LYVE1^–^CD31^+^ ECs were FACS-sorted from LPS-stimulated intestinal lamina propria with and without macrophage depletion and subjected to mRNA sequencing. (**A**) Principal component analysis comparing macrophage-intact/-depleted (MΦ-intact/MΦ-depletion) conditions based on differentially expressed genes (DEGs). (**B**) Gene ontology (GO) pathway enrichment analysis of DEGs. The top 20 enriched terms are shown. (**C**) Expression heatmaps of chemokines and (**D**) cellular adhesion molecules (CAMs) relevant to PMNs indicate macrophage priming of EC responses. Color scales represent percentage change in gene expression. (**E**–**H**) CSF-1R Ab–mediated macrophage depletion in control or LPS-stimulated CX3CR1-EGFP mice. To visualize and quantify EC ICAM-1 expression, a low dose of fluorescently labeled anti–ICAM-1 Ab (2 μg, i.v.) was used (red). (**E**) Representative whole-mount confocal microscopy images show VAM localization to high ICAM-1 regions with LPS stimulation and loss of local ICAM-1 enrichment with macrophage depletion. Scale bar: 20 µm. (**F**) Quantification of ICAM-1 expression normalized to PECAM-1 staining to account for tissue depth variation. (**G**) Quantification of the relative ICAM-1 expression per 25 μm vessel segments with and without macrophage contact. (**H**) Comparison of the relative ICAM-1 expression per 25 μm vessel segments with and without macrophage depletion. Dotted region highlights the loss of ICAM-1 hot spots. (**I**) Quantification and (**J**) representative flow diagram of mucosal EC ICAM-1 expression by flow cytometry. (**K**) Representative flow diagrams and (**L**) quantification of ICAM-1 expression in cultured mouse (bEnd.3) and human (HUVEC) ECs, respectively, treated with conditional media from murine BM-derived and human THP-1 monocytic cell line, differentiated with IFN-γ/LPS so that they resemble tissue inflammatory macrophages. For whole-mount preparations, images are representative of *n* = 3–5 mice, with each data point representing a field of view. For flow cytometry, *n* = 3–4 independent experiments. Spnt, supernatant. *P < 0.05, **P < 0.01, ***P < 0.001. Two-sided Student’s *t* test and 1-way ANOVA with Tukey’s multiple comparison test. Data are presented as mean ± SEM.

We next hypothesized that VAMs may prime EC activation to support more efficient PMN TEM. To test this, CD45^–^LYVE1^–^CD31^+^ ECs were FACS-sorted from LPS-stimulated, collagen-digested intestinal lamina propria with and without macrophage depletion and subjected to 3′-mRNA sequencing. A 3D principal component analysis of differentially expressed genes (DEGs) clearly separated ECs with and without macrophage depletion, indicating a significant effect of macrophages on the EC transcriptional program (Figure 2A). DEG analyses identified 543 upregulated and 693 downregulated genes between the two conditions (Supplemental Figure 2A). Extended gene ontology (GO) network analyses (using Metascape) focused on the potential macrophage effect on EC function, revealed overrepresentation of terms associated with cytokine production, cell adhesion, and actin cytoskeletal organization with macrophage depletion (Figure 2B). All the above biological processes are required for PMN TEM. Quantification of DEG fold change following macrophage depletion revealed a robust reduction in EC cytokine production as well as downregulation of several major EC adhesion molecules, including ICAM-2 and to a lesser degree ICAM-1 (shown as percentage in Figure 2, C and D). Macrophage depletion similarly led to overall downregulation of genes encoding many EC junctional components (claudins, cadherins, gap junctions) and intercellular adhesion proteins (integrins), as well as an induction of proapoptotic caspases, indicating altered/weakening of the EC integrity (Supplemental Figure 2, B–D).

### VAMs promote localized ICAM-1 upregulation in inflamed intestinal ECs.

Expression of both ICAM-2 and ICAM-1 is induced following EC activation (31–33), and both molecules contribute to the regulation of PMN recruitment. We thus used in situ fluorescence labeling and whole-mount confocal microscopy to examine expression and distribution patterns of these adhesion molecules in inflamed ECs.

In these experiments, blood vessels in CX3CR1-EGFP mice were costained for ICAM-2 or ICAM-1 and PECAM-1 (using function nonblocking Abs, 2 μg, i.v.). PECAM-1 was used as reference protein to normalize for depth variations during confocal image acquisition (27). Fluorescence analyses as an index of protein expression revealed no significant change in EC ICAM-2 expression with macrophage depletion (Supplemental Figure 2, E–G). In contrast, macrophage depletion significantly dampened LPS-induced EC ICAM-1 upregulation (Figure 2, E and F). Importantly, we noted the appearance of distinct high ICAM-1 expression regions in inflamed vessels, with more than 85% of VAMs specifically localizing to these regions (Supplemental Figure 2H and Figure 2E, representative images). Indeed, expression analyses revealed distinct enrichment of ICAM-1 specifically in regions of VAM-EC contact, forming ICAM-1 hot spots (Figure 2G). Further partitioning of the vessel wall into 25 μm–long segments, capturing regions of VAM-EC contact and ICAM-1 hot spots, revealed substantial loss of initial heterogeneity in the ICAM-1 expression patterns and specifically that of ICAM-1 hot spots with macrophage/VAM depletion (Figure 2H). These data indicate VAM involvement in ICAM-1 hot spot formation. Macrophage regulation of ICAM-1 expression was similarly confirmed by flow cytometry of digested intestinal lamina propria, where LPS induced ICAM-1 but not ICAM-2 upregulation in CD45^–^LYVE1^–^CD31^+^ ECs was suppressed with macrophage depletion (Figure 2, I and J, and Supplemental Figure 2I).

Next, in vitro cultures were used to directly test macrophage regulation of EC ICAM-1. In these experiments, cultured mouse (bEnd.3) and human (HUVEC) ECs were treated with conditioned media from the respective murine BM-derived or human THP-1 monocytic cell line, differentiated in vitro with IFN-γ/LPS to resemble tissue inflammatory macrophages. For both murine and human cells, macrophage-conditioned media significantly induced EC ICAM-1 expression compared with IFN-γ/LPS containing culture media alone (Figure 2, K and L, and Supplemental Figure 3, A and B). LPS removal from conditioned media (Pierce High-Capacity Endotoxin Removal Resin, Thermo Fisher Scientific, 88273) did not affect the induction in EC ICAM-1 expression (Supplemental Figure 3, C and D), confirming macrophage regulation of EC ICAM-1 via soluble factor(s) release.

### Macrophages upregulate EC ICAM-1 via the release of TNF-α.

**Figure 3:**
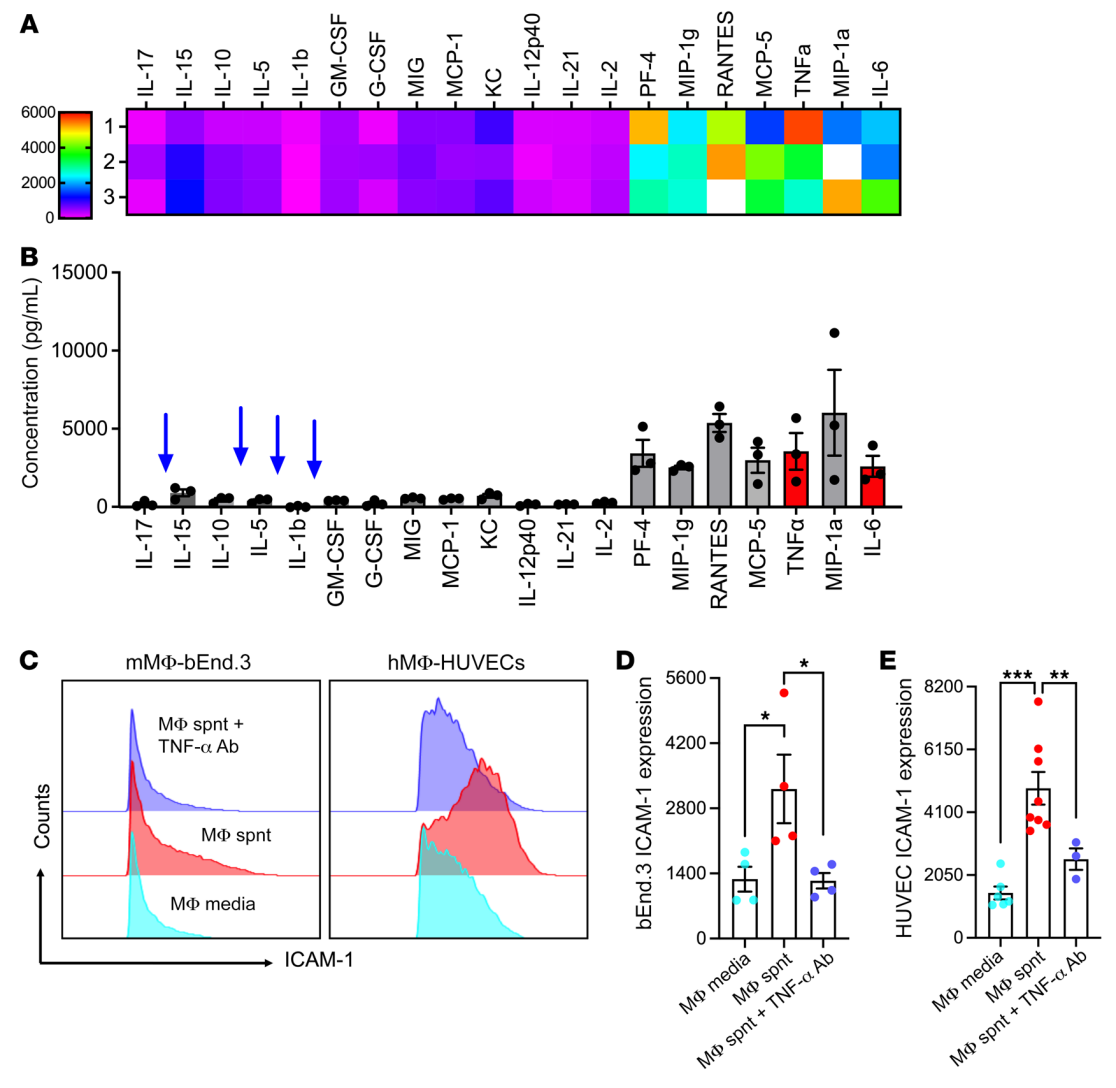
Macrophages upregulate EC ICAM-1 via the release of TNF-α. (**A** and **B**) Conditioned media from murine IFN-γ/LPS-differentiated BM-derived macrophages was subjected to targeted (40-target) cytokine array. (**A**) Heatmap and (**B**) detected concentration of the most highly expressed cytokines. (**C**–**E**) Cultured murine (bEnd.3) and human (HUVEC) ECs were treated with BM-derived and human THP-1 monocytic cell line conditioned media, respectively, with and without cotreatment with anti–TNF-α inhibitory Abs. (**C**) Representative flow diagram and (**D** and **E**) quantification of EC ICAM-1 expression. Cytokine array data are representative of 3 independent experiments. For flow cytometry, *n* = 4–8 independent experiments. Spnt, supernatant. *P < 0.05, **P < 0.01, ***P < 0.001. One-way ANOVA with Tukey’s multiple comparison test. Data are presented as mean ± SEM.

To identify macrophage-derived factors driving EC ICAM-1 upregulation, supernatants collected from BM-derived, IFN-γ/LPS-differentiated macrophages (24 hours following rigorous wash to remove IFN-γ/LPS) were profiled by a 40-target inflammatory cytokine protein. TNF-α and IL-6, both of which have been reported to regulate ICAM-1 expression, were identified among top hits (Figure 3, A and B). Other cytokines known to regulate ICAM-1 expression, including IL-5, IL-17, IL1b, and GM-CSF, were either not or minimally detected in macrophage-conditioned media. Functional ex vivo studies using inhibitory Abs for both murine and human EC-macrophage-conditioned media cultures revealed that neutralization of TNF-α but not IL-6 suppressed ICAM-1 upregulation (Figure 3, C–E, and Supplemental Figure 3, E and F). Consistent with the lack of effect of macrophages on ICAM-2 expression in vivo, macrophage-conditioned media failed to induce EC ICAM-2 expression, and inhibition of TNF-α similarly had no significant impact (Supplemental Figure 3G). These data suggest that inflamed macrophages via TNF-α release can prime ECs to upregulate ICAM-1.

### Macrophages regulate PMN adhesion and TEM via TNF-α–dependent EC ICAM-1 induction.

**Figure 4:**
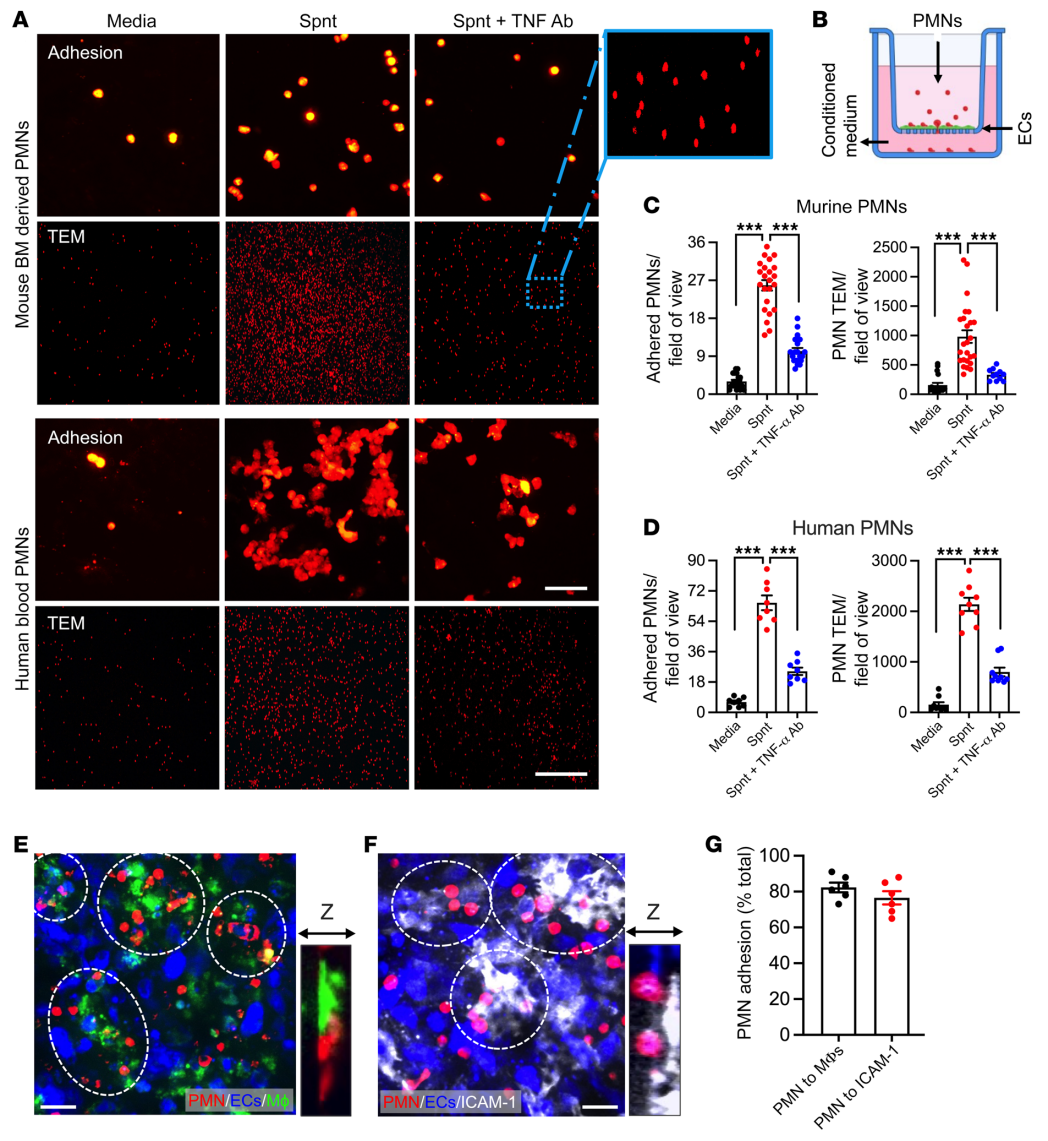
Macrophages regulate PMN adhesion and TEM via TNF-α–dependent EC ICAM-1 induction. (**A**–**D**) Murine/human PMN adhesion and TEM across murine bEnd.3 or human HUVECs were examined using a Transwell setup. Murine BM-PMNs and human blood PMNs were fluorescently stained using Cell Tracker Orange, stimulated with fMLF (500 nM and 200 nM, respectively, 10 min), and were induced to adhere/migrate across ECs by introducing BM-derived or human THP-1 cell–conditioned media with and without Ab TNF-α neutralization against the bottom chamber. (**A**) Representative images reveal decreased PMN adhesion (PMNs on filters) and TEM (depicting PMNs that have migrated to the bottom chamber) with TNF-α inhibition. Scale bars: 20 μm (adhesion); 100 μm (TEM). The zoom-in region shown in the dotted box is a ×10 magnification of the original image. (**B**) Schematic depicting the experimental setup. (**C**) Quantification of murine and (**D**) human PMN adhesion and TEM. (**E**–**G**) A triple coculture of PMN, EC, and macrophages was used, where LPS/IFN-γ–stimulated CX3CR1-EGFP (green) macrophages washed of remanence of stimulation media were added to the basal side of cultured confluent EC monolayers (inverted orientation) followed by Cell Tracker Orange–labeled PMN addition to the apical side (top chamber). (**E**) Representative *Z*-stack projection (~40 μm) image shows localized PMN (red) attachment to ECs (blue) in regions of macrophage (green) contact (highlighted by white dotted circles). Zoom-in image depicts close PMN-macrophage contact. Sale bar: 20 μm. (**F**) Representative *Z*-stack projection images of ICAM-1–stained EC monolayers (following PMN attachment) show preferential PMN binding to high EC ICAM-1 expression regions. Zoom-in image depicts PMN binding to EC ICAM-1. Scale bar: 20 μm. (**G**) Quantification for PMN adhesion frequency to regions of macrophage contacts and regions enriched for ICAM-1 expression. *n* = 4 independent experiments in duplicates per condition. ***P < 0.001. One-way ANOVA with Tukey’s multiple comparison test. Data are presented as mean ± SEM.

We next asked if macrophage TNF-α–dependent induction of ICAM-1 directly drives PMN adhesion and TEM. To test this, we utilized a Transwell system, where murine bEnd.3 cells or human HUVECs were cultured until confluency on permeable supports with 0.4 or 3 μm pore size to test PMN adhesion and TEM, respectively (34). ECs were stimulated with macrophage-conditioned media added to the bottom chamber (24 hours, as in cytokine studies, Figure 3A) with or without inhibitory Abs against TNF-α (10 μg/mL) to promote or inhibit ICAM-1 upregulation. Freshly isolated BM-derived murine or human peripheral blood PMNs (5 × 10^5^/well) were fluorescently labeled (Cell Tracker Orange) for visualization and seeded in the top chamber. Adhesion to ECs (following 1-hour incubation) or PMN TEM (4 hours) was quantified by imaging of the permeable supports or bottom chambers, respectively (Figure 4, A and B). Both murine and human macrophage-conditioned media promoted PMN adhesion and TEM, whereas TNF-α neutralization in macrophage supernatants abrogated this effect (Figure 4, C and D), consistent with the observed suppression of ICAM-1 upregulation (Figure 3C).

Finally, confocal microscopy imaging of PMN-EC-macrophage cocultures on permeable supports confirmed enrichment of PMN-EC adhesive interactions specifically at macrophage contact regions, while also confirming localization to regions of high ICAM-1expression, consistent with in vivo observations in inflamed intestines (Figure 4, E–G).

### Inflamed intestinal ECs express TNFR2 to interact with VAM-derived TNF-α.

**Figure 5:**
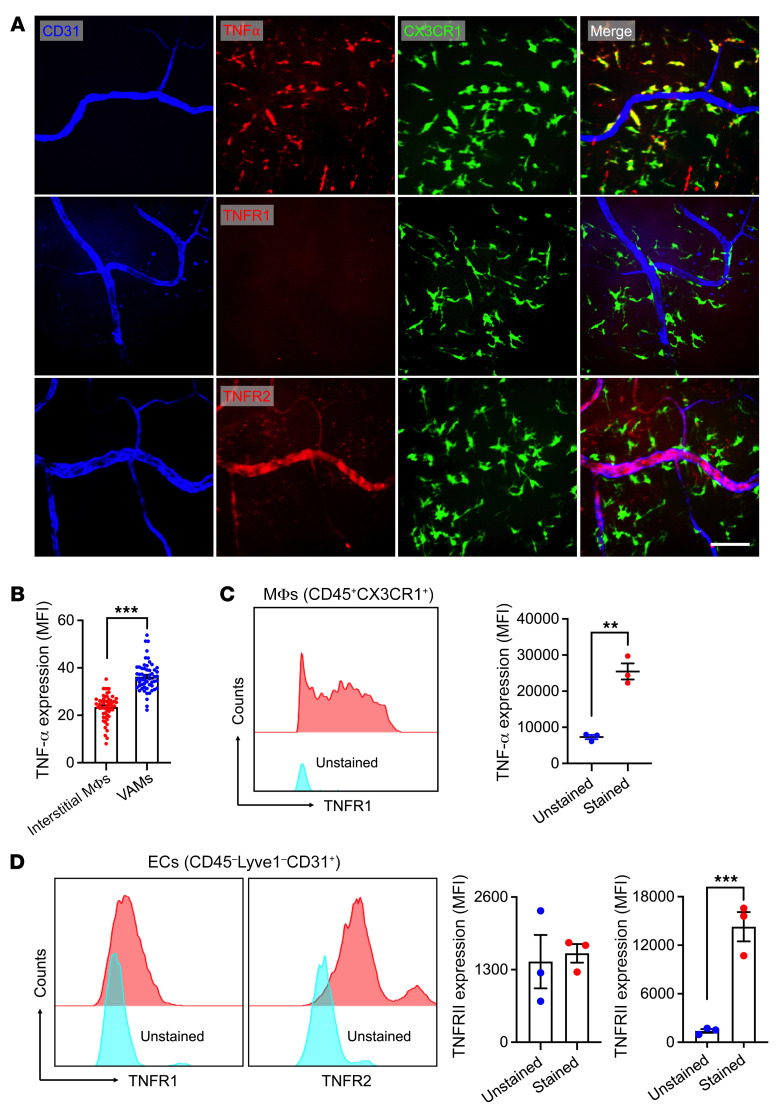
Inflamed intestinal ECs express TNFR2 to interact with VAM-derived TNF-α. (**A** and **B**) Combined in situ and whole-mount staining and confocal microscopy was performed on CX3CR-EGFP reporter mice to examine expression of TNF-α and its receptors TNFR1 and TNFR2. (**A**) Representative images show TNF-α expression (red) by interstitial macrophages (elevated specifically in VAMs). TNFR2 but not TNFR1 is expressed by gut ECs. Scale bar: 25 μm. (**B**) Quantification of mean fluorescence intensity (MFI) in interstitial macrophages remote from vessels and in VAMs. (**C** and **D**) Flow cytometry–based analyses were performed on LPS-stimulated, digested intestinal mucosa. (**C**) Quantification of TNF-α expression in CD45^+^CX3CR1^+^ gut macrophages and (**D**) TNFR1/TNFR2 in CD45^–^LYVE1^–^CD31^+^ ECs. For whole-mount preparations, images are representative of *n* = 4 independent experiments. Each data point represents a field of view. For flow cytometry, *n* = 3 independent experiments. **P < 0.01, ***P < 0.001. Two-sided Student’s *t* test. Data are presented as mean ± SEM.

To ascertain if these findings were recapitulated in vivo, in situ fluorescence labeling and confocal microscopy were used to examine the expression of macrophage TNF-α and major EC TNF-α receptors (TNFR1 and TNFR2) in inflamed gut. In these experiments, CX3CR1-EGFP mice were stimulated with LPS (100 μg, 24 hours i.p.), and Abs against either TNFR1 or TNFR2 were coinjected with CD31 to specifically label EC TNFRs and to outline the vasculature (i.v. 1 hour prior to euthanasia). Macrophage/VAM TNF-α staining was performed directly on excised intestinal segments. Whole-mount confocal imaging revealed that TNF-α was expressed by approximately 90% of tissue CX3CR1^+^ macrophages (Figure 5A, top). Importantly, VAMs expressed significantly higher TNF-α levels relative to interstitial macrophages (~1.8-fold, Figure 5B), supporting the notion of TNF-α–dependent EC priming by VAMs. Interestingly, inflamed intestinal ECs expressed TNFR2 but not TNFR1, as it colocalized with CD31-costained vessels (Figure 5A, middle and bottom). TNF-α expression by CD45^+^CX3CR1^+^ intestinal macrophages and the expression of TNFR2 but not TNFR1 by inflamed CD45^–^LYVE1^–^CD31^+^ ECs was also confirmed by flow cytometry of digested intestinal mucosa (Figure 5, C and D). These observations indicate that VAM-derived TNF-α may bind EC TNFR2 to induce upregulation of ICAM-1.

### Macrophage TNF-α and EC TNFR2 axis regulates PMN TEM.

**Figure 6:**
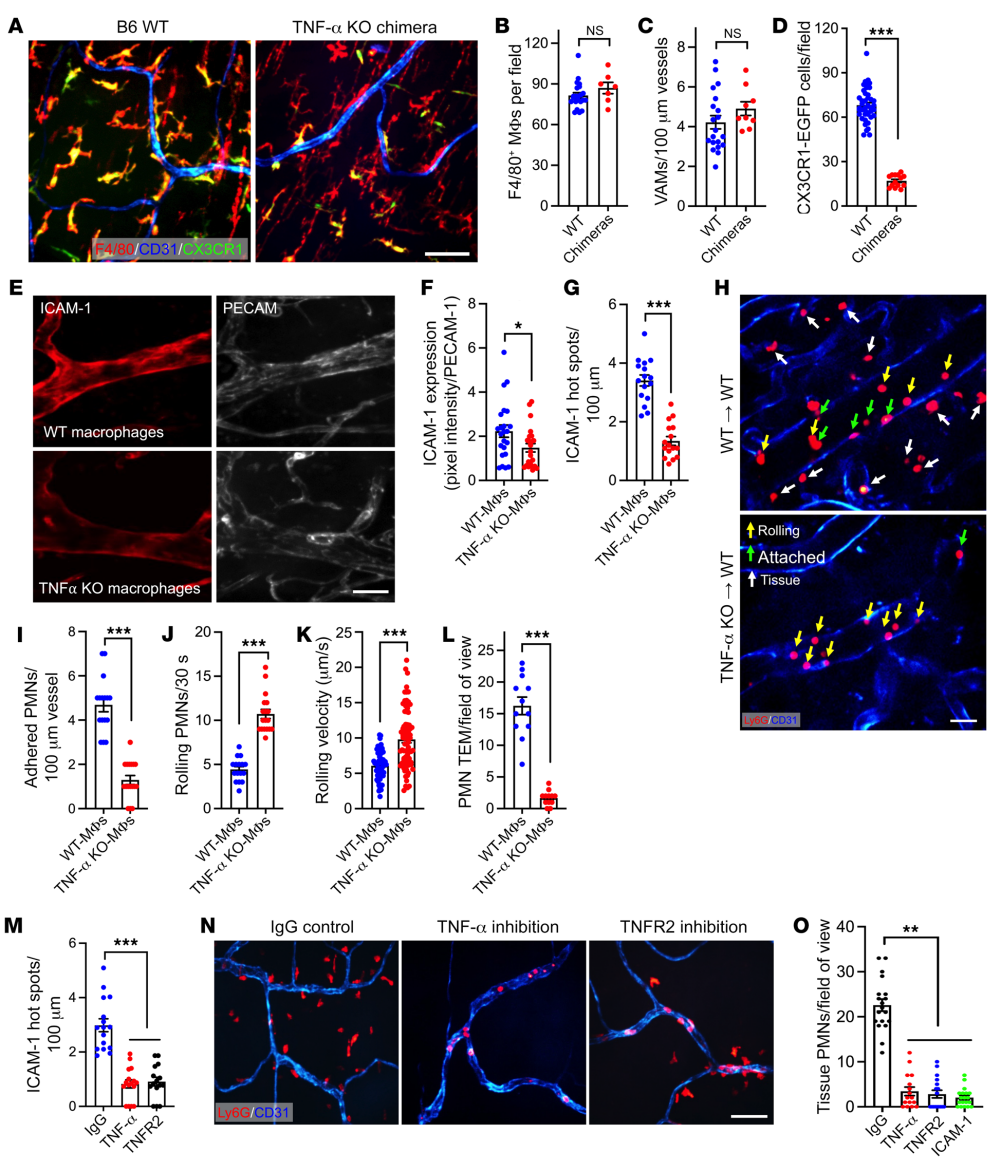
Macrophage TNF-α and EC TNFR2 axis regulates PMN TEM. (**A**–**D**) TNF-α–KO or control WT macrophage chimeras were generated to test whether macrophage-derived TNF-α promotes PMN TEM. (**A**) Representative whole-mount confocal microscopy images show near-complete loss of host CX3CR1 macrophages and repopulation by donor (nonfluorescent, F4/80^+^) macrophages 8 weeks following grafting. Scale bar: 25 μm. (**B**) Quantification of total (F4/80^+^) repopulating donor macrophages, (**C**) VAMs per vessel length, and (**D**) reduction in host CX3CR1 macrophages 8 weeks following grafting. (**E**) Representative whole-mount confocal microscopy images and (**F**) quantification, showing reduced ICAM-1 expression (in situ fluorescence labeling, red) relative to PECAM-1 (CD31, blue) in TNF-α–KO macrophage chimeras. Scale bar: 20 µm. (**G**) Quantification of ICAM-1 hot spots per vessel length in TNF-α–KO and WT macrophage chimeras. (**H**) Representative time lapse intravital microscopy (IVM) images show decreased rolling (yellow arrows) and increased PMN adhesion (green arrows) in WT chimeras, whereas PMN adhesion was substantially reduced in TNF-α–KO macrophage chimeras. Scale bar: 20 μm. (**I**) Quantification of PMN adhesion from IVM, (**J**) PMN rolling (per 30 seconds recordings), (**K**) rolling velocity, and (**L**) extravasated tissue PMNs (white arrows in **H**). (**M**–**O**) WT mice were pretreated with IgG control or neutralizing Abs against TNF-α (400 μg, i.p.), TNFR2, or ICAM-1 (400 μg, i.v.). (**M**) Quantification of ICAM-1 hot spots per vessel length using in situ fluorescence labeling. (**N**) Representative whole-mount confocal microscopy images and (**O**) quantification, showing a reduced number of extravasated PMNs with TNF-α/TNFR2/ICAM-1 neutralization (PMNs stained for Ly6G, red). Scale bar: 25 μm. For whole-mount preparations, images are representative of *n* = 6 independent experiments. For IVM, *n* = 3–5 mice per condition. *P < 0.05, **P < 0.01, ***P < 0.001. Two-sided Student’s *t* test and 1-way ANOVA with Tukey’s multiple comparison test. Data are presented as mean ± SEM.

We next generated TNF-α–KO macrophage chimeras to test whether macrophage-derived TNF-α triggered localized formation of ICAM-1 hot spots to facilitate PMN TEM. In these experiments, BM from TNF-α–KO or control WT mice was grafted into irradiated CX3CR-EGFP recipients, and 8 weeks following grafting whole-mount confocal and intravital microscopy was performed to quantify ICAM-1 expression patterns and PMN-EC interactions. We determined that 8 weeks were required to repopulate host gut with donor macrophages. At this time, the total number of tissue macrophages and VAMs (stained for F4/80) was not significantly different between WT and TNF-α–KO chimeric mice; however, more than 85% of host CX3CR-EGFP macrophages were replaced with grafted non-EGFP cells (Figure 6, A–D). A detailed time course of tissue repopulation by grafted macrophages is shown in Supplemental Figure 4, A–D. Of note, even 8 weeks after grafting, a residual (<15%) host CX3CR-EGFP cell population has remained, likely representing the self-renewing gut macrophage population (15).

In situ fluorescence labeling of ICAM-1 and whole-mount confocal imaging revealed an overall reduction in EC ICAM-1 expression and, more importantly, significant decrease in the number of ICAM-1 hot spots in LPS-stimulated TNF-α–KO macrophage compared with WT macrophage grafted chimeras (Figure 6, E–G). Consistently, intravital imaging of TNF-α–KO chimeras revealed significantly reduced PMN adhesion (~3.6-fold), whereas the number of rolling PMNs was increased (>2-fold) with elevated rolling velocities (Figure 6, H–K, and Supplemental Videos 3 and 4) following LPS stimulation. Consistent with impaired adhesion, the number of extravasated PMNs in TNF-α–KO chimeras was also significantly reduced (Figure 6L).

To further confirm the TNF-α–TNFR2 axis contribution to ICAM-1 hot spots formation and PMN TEM, mice were treated with either control IgG or inhibitory anti–TNF-α (400 μg, i.p., to neutralize interstitial/VAM-derived TNF-α) or anti-TNFR2 (400 μg, i.v., to neutralize EC TNFR2) Abs. Whole-mount and intravital imaging revealed that both treatments, but not IgG control Ab, significantly reduced the overall EC ICAM-1 expression and the number of ICAM-1 hot spots (Supplemental Figure 4E and Figure 6M). Consistently, PMN tissue extravasation was also significantly reduced with both treatments (Figure 6, N and O). An overall reduction in EC ICAM-1 expression was confirmed by flow cytometry (Supplemental Figure 4F). Ab-mediated inhibition of ICAM-1 (400 μg, i.v.) similarly suppressed PMN TEM (Figure 6O and Supplemental Figure 4G), consistent with the idea that VAM-facilitated TNF-α/TNFR2 signaling regulates PMN TEM via localized generation of ICAM-1 hot spots.

### Evidence of VAM recruitment and VAM-EC-PMN interactions in clinical IBD specimens.

**Figure 7:**
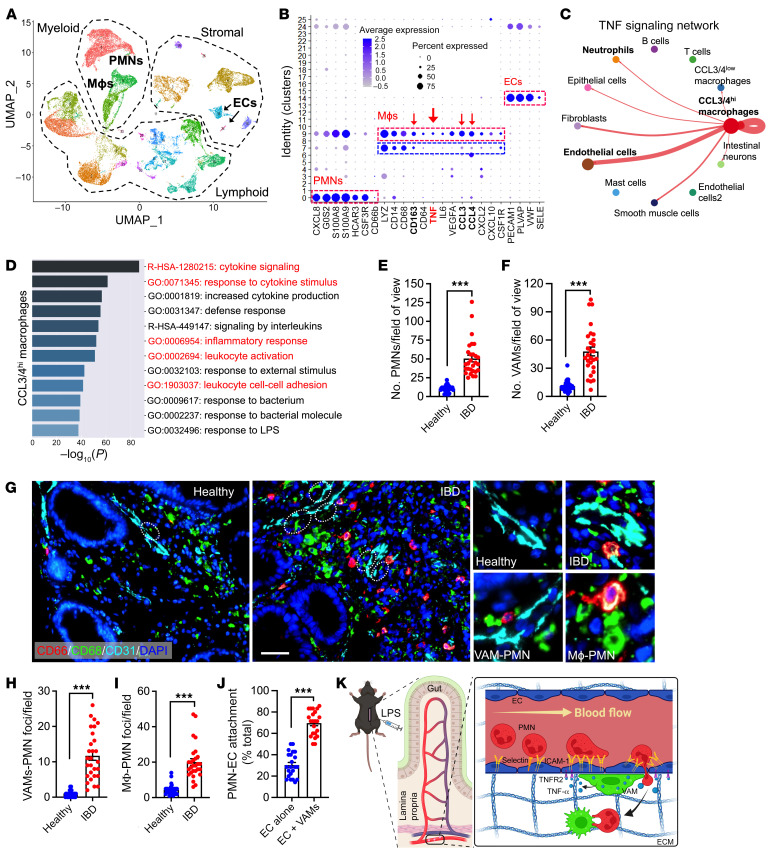
Evidence of VAM recruitment and VAM-EC-PMN interactions in clinical IBD specimens. (**A**–**D**) Single-cell RNA-Seq was performed on biopsies from patients with active UC. (**A**) Uniform manifold approximation and projection (UMAP) analyses of integrated data from 4 patients with IBD. (**B**) Dot plot showing scaled expression of selected signature genes for PMN, macrophage, and EC clusters. Gene expression in each cluster was scaled across all clusters. Dot size represents the percentage of cells in each cluster, and color indicates expression (number of reads). (**C**) Outgoing communication pattern analyses by CellChat, specifically focused on network centrality analysis of inferred TNF-α signaling with macrophages defined as senders. Interaction strengths are shown/scaled between annotated seurat clusters. (**D**) GO analysis of DEGs for the CCL3/4^hi^ macrophage cluster. The top 12 GO enrichment terms are shown. Analyses were performed with a Fisher’s exact test, with *P* < 0.01. Terms shown in red highlight more relevant terms consistent with observations made in a murine model. (**E**–**J**) Multiplex immunofluorescence staining was performed on healthy biopsied tissue and biopsied tissue from patients with active UC (*n* = 4 patient/conditions). (**E**) Quantification from multiplex imaging of PMN tissue infiltration and (**F**) VAM numbers. (**G**) Representative multiplex immunofluorescence images. White dotted circles highlight VAMs. Scale bar: 50 μm. Zoom-in panels (on the right) depict healthy and inflamed vessels with respective recruitment of VAMs. PMNs leaving the vessels specifically at a region of VAM contact and PMN-macrophage interactions in the interstitial are shown. (**H** and **I**) Quantification of VAM and interstitial macrophage interactions with PMNs and (**J**) the frequency of PMN-EC interactions specifically at regions of EC-VAM contact or ECs remote from VAMs (ECs alone). For all image analyses, 5–8 images per patient were analyzed. Each data point represents a field of view. (**K**) Representation schematic summarizing the mechanistic VAM regulation of EC function and PMN TEM. ***P < 0.001. Two-sided Student’s *t* test. Data are presented as mean ± SEM.

To establish the clinical relevance of VAM-mediated EC priming and regulation of PMN TEM in inflamed gut, biopsied tissue from 4 patients with ulcerative colitis (UC) was analyzed by single-cell RNA-Seq to examine transcriptional programs involved in macrophage-EC-PMN crosstalk. Rigorous quality controls are detailed in the methods section (Supplemental Figure 5A). Following integration, 47, 688 cells were clustered into 26 different clusters using principal component analysis followed by uniform manifold approximation/projection under Louvain’s algorithm (Figure 7G). Expression of DEGs for each cluster is shown in Supplemental Figure 5B. PMN (*CXCL8*, *S100A8*, *CSF3R*), EC (*PECAM1*, *PLVAP*, *VWF*), and macrophage (*LYZ*, *CD68*, colony stimulating factor 1 receptor [*CSF1R*]) populations were identified via common lineage markers (annotated, Figure 7A and Supplemental Figure 5C). This was further validated using the online databases Human Primary Cell Atlas and panglaoDB. Two major macrophage populations were identified, clusters 9 and 7, expressing high and low levels of activation/inflammatory polarization-associated chemokines CCL3 and CCL4, respectively. CCL3/CCL4^hi^ macrophages (both genes were also associated with VAMs) were also enriched with additional VAM-associated genes such as *VEFGA*, *CD163*, and *CXCL2* (Figure 7B) (35, 36). Importantly, the CCL3/CCL4^hi^ macrophage cluster also showed elevated TNF-α expression (Figure 7B), and CellChat network centrality analyses (37) inferred outgoing TNF-α signaling mainly focused onto ECs and to a lesser degree to PMNs (Figure 7C). Specificity of the macrophage-EC crosstalk was indicated by CellChat analyses centered on IL-1 signaling, revealing CCL3/CCL4^hi^ macrophages primarily communicating with PMNs and CCL signaling linking intestinal neurons and ECs (Supplemental Figure 5, E and F). Extended GO analyses identified enrichment of terms involved in cytokine signaling response, leukocyte activation, and cell-cell adhesion for CCL3/CCL4^hi^ but not the CCL3/CCL4^lo^ macrophage cluster (Figure 7D and Supplemental Figure 5D).

Finally, consistent with clinical features of active IBD, multiplex immunofluorescence (IF) staining of healthy and active IBD biopsy specimens revealed a robust PMN recruitment and accumulation in diseased tissue (Figure 7, E and G). Supporting our murine and human single-cell data, the number of VAMs in inflamed colon (Figure 7, F and G) as well as VAM-PMN and interstitial macrophage-PMN interactions were similarly increased in tissue with active disease as compared with healthy colon tissue (Figure 7, H and I). Importantly, as we have found in an animal model, approximately 70% of all PMNs-EC interactions occurred in regions of VAM-EC contacts (Figure 7J), Thus, unbiased transcriptome and IF assessment of macrophage localization and function in IBD supports the idea that a macrophage subset may act as VAMs to prime EC responses via cytokine release to accommodate PMN TEM in inflamed colon mucosa (summarized by the schematic of experimental model, Figure 7K).

## Discussion

PMNs play important roles in host defense and restoration of tissue homeostasis; however, their activity in tissue also underlies a variety of pathological conditions, including IBD, acute lung injury, cardiovascular disease, rheumatoid arthritis, and atherosclerosis (38–41). Importantly, exacerbated inflammation and tissue damage associated with PMN activity primarily results from excessive PMN accumulation in specific tissue locations, following their crossing of the vascular wall. Therefore, insights into the regulatory mechanisms of PMN trafficking including TEM and interstitial migration are of potential clinical and therapeutic relevance for many inflammatory disorders.

Circulating PMNs upon detection of inflammatory insult navigate toward affected tissue regions. Such recruitment cascade initiates with activation of both PMNs and ECs ultimately leading to PMN TEM. While adhesion molecules and signaling involved in this process have been extensively studied (4, 42), early events driving EC activation are not known. The current study investigated this less understood part of the PMN recruitment cascade, focusing on early initiation mechanisms of EC priming to support PMN TEM. We found that, in inflamed mucosa, VAMs serve as gatekeepers to initiate and facilitate PMN TEM. Via the release of TNF-α, which acted on TNFR2, VAMs triggered localized induction in EC ICAM-1, forming ICAM-1 hot spots to promote PMN-EC adhesive contacts and PMN TEM. Although TNF-α–driven upregulation of ICAM-1 may not be surprising, our observations of ICAM-1 hot spots formation indicate localized priming of ECs and spatially different PMN responses with potential functional implications. We found that the number of VAMs in inflamed mucosa was significantly increased. Whether interstitial macrophages are actively recruited to associate with blood vessels in inflamed mucosa and the underlying mechanisms that may mediate such recruitment is a topic that we are actively pursuing. Similarly, given macrophage plasticity and functional subletting (43–48), whether VAMs represent a unique subset of interstitial macrophages remains to be determined. Indeed, in the absence of mucosal VAM specific markers, a limitation of our study is the utilization of an anti–CSF-1R Ab for depletion, which targets all mucosal macrophage populations rather than specifically depleting VAMs. We currently optimizing approaches to define specific VAM markers in hope of addressing this limitation in future studies.

Depletion of interstitial macrophages and VAMs robustly reduced LPS-induced PMN adhesion, reducing PMN TEM and accumulation in inflamed mucosa. These experiments established the importance of macrophage contribution to the regulation of PMN TEM. Subsequent total input mRNA sequencing of inflamed intestinal ECs with and without macrophage depletion revealed macrophage contributions to EC priming and activation. GO analyses implicated macrophages in maintenance of EC junctional integrity and survival, as has been previously suggested in other tissues (7–9). Importantly, macrophage removal significantly altered EC transcriptional programs of cytokine/chemokine production, actin cytoskeletal organization and adhesion molecule expression, all of which critically contribute to PMN TEM regulation.

Intravital imaging of live intestines revealed a robust enrichment of ICAM-1 in VAM-EC contact areas, creating preferred regions for PMN TEM. Indeed, our group and others have previously demonstrated that PMN TEM is not a uniform process but takes place in specific locations, termed hot spots (49–51), featuring high levels of ICAM-1 expression (52, 53). Macrophage/VAM depletion eliminated these ICAM-1 hot spots, confirming their contribution. Similarly, supernatants from LPS/IFN-γ–stimulated BM-derived murine or human macrophages induced a robust upregulation of ICAM-1 in both murine and human ECs, indicating that macrophages via the release of soluble factor(s) stimulate EC activation and ICAM-1 induction. Indeed, a screen of protein content in macrophage supernatants identified several potential cytokines to mediate EC ICAM-1 induction, of which via function inhibitory studies, we established TNF-α to be the major contributor. Subsequent in vivo experiments using chimeric macrophage-specific TNF-α–KO animals or Ab inhibition of EC TNFRs confirmed macrophages as a major contributor, however, they were likely not the only source of TNF-α to promote EC ICAM-1 upregulation. Although ECs are known to express both TNFR1 and TNFR2, which trigger both shared and distinct downstream signaling (54), IF confocal imaging revealed that inflamed intestinal ECs mainly expressed TNFR2. TNFR2 binds with higher affinity to the membrane-bound form of the cytokine as compared with TNFR1 (55), supporting the idea of local cytokine transfer from VAMs (expressing higher TNF-α levels than interstitial macrophages) to ECs. Consistent with the idea of TNFR2 regulating PMN TEM, its depletion reduced PMN tissue infiltration (56).

Finally, using multiplex IF, we confirmed recruitment of VAMs and formation of VAM-EC-PMN contacts in clinical samples from patients with IBD. Further supporting the clinical relevance of our observations, single-cell analyses of IBD tissue identified a macrophage population enriched in previously reported VAM genes. These cells were enriched in intercellular communication governing TNF-α signaling directed from this macrophage subset toward ECs. This communication pattern was overrepresented by genes encoding cellular activation and cytokine production, including TNF-α, as well as in genes involved in the regulation of cell-to cell adhesions and leukocyte activation. These observations support our murine mechanistic studies and perhaps indicate an activated/inflammatory nature of VAMs. In summary, our studies identify what we believe to be a novel role of interstitial macrophages/VAMs in locally priming gut ECs to facilitate PMN recruitment. The goal of most current therapeutic interventions, including these in IBD, is achieving homeostatic restoration and tissue healing. Given the frequent association of PMN presence with tissue injury and worsened clinical outcomes, targeting a specific macrophage population and activity may offer therapeutic opportunities to limit PMN tissue accumulation and limit PMN-driven disease pathologies. Our studies also highlight an additional mechanism of action of the anti–TNF-α therapies, commonly used in inflammatory diseases, to limit inflammatory EC activation.

## Methods

Please refer to Supplemental Methods for fully detailed experiential protocols and procedures.

### Animals and cell lines.

C57BL/6J and TNF-α–KO mice (B6.129S-Tnftm1Gkl/J) were purchased from The Jackson Laboratories. CX3CR1-EGFP reporter mice were a gift from H. Perlman (Department of Medicine, Northwestern University Feinberg School of Medicine, Chicago, Illinois, USA) (57).

### Cell lines.

Microvascular brain ECs (bEnd.3), HUVECs, and the human monocytic cell line THP-1 were cultured the manufacturer’s instructions (ATCC). Murine PMNs were isolated from BM and enriched to approximately 90% purity using Histopaque gradients as described previously (58, 59). Mouse BM-derived macrophages were isolated and differentiated as described previously (60). Human PMNs were isolated from healthy blood by density gradient centrifugation (3, 61).

### Intravital imaging.

Intravital imaging was performed on exposed segments of the small intestine in anesthetized mice, using an Olympus BX-51WI Fixed-Stage illuminator equipped with a Yokogawa CSU-X1-A1 spinning disk (62). To visualize blood vessels and analyze adhesion molecule expression, primary labeled anti–ICAM-1/2 Abs (clones YN1/1.7.4 and 3C4) with and without nonblocking anti–PECAM-1 Ab (clone 390) conjugated to DyLight−647 for plane normalization (27) were injected retro-orbitally. In whole-mount preparations following image acquisition, fluorescence intensity levels, as an index of protein expression, were analyzed by projecting a line 3-pixels wide along the wall at the central plane of the vessel cross section and obtaining the intensity profile along that line. PMN rolling adhesion and TEM were analyzed from recordings of random fields containing 30–50 μm venules (62). BM chimeras were generated per standard protocol (63, 64). Reconstitution was confirmed by IF for CD45^+^ immune cells and F4/80^+^ macrophages and absence of host-EGFP^+^ tissue-resident cells.

### Flow cytometry and immunofluorescence labeling.

BD LSR Fortessa X and FlowJo 10.7 software 20 (BD) were used for flow cytometry analyses (65). For multiplex IF staining, unconjugated CD31 (Abcam, clone EPR3131), CD66b (Novus Biologicals, clone G10F5), and CD68 (Dako Agilent, clone PG-M1) and secondary Alexa fluorophore–conjugated Abs were used and validated as described previously (66). Multiplex panel runs were executed in the Lunaphore COMET platform. For image analyses, slides were automatically scanned using the Lunaphore COMET. At least *n* = 8 regions per sample were captured. Cells of interest were quantified using ImageJ software (NIH).

### RNA-Seq.

Bulk RNA-Seq on FACS-sorted murine CD45^–^Lyve1^–^CD31^+^ ECs and single-cell RNA-Seq of colonic biopsies from patients with UC were performed and analyzed as detailed in Supplemental Methods. cDNA library construction and RNA-Seq were conducted at the Northwestern University sequencing core facility using an Illumina HiSeq 4000 NGS or the Illumina NovaSeq 6000 systems (Illumina).

### Statistics.

Statistical significance was assessed by 2-tailed Student’s *t* test or by 1-way ANOVA with Newman-Keuls multiple-comparison test using Graphpad Prism (v4.0). Normal data distribution was evaluated by the Shapiro-Wilk test. Statistical significance was set at *P* < 0.05. All data are shown as ± SEM.

### Study approval.

All human studies were reviewed and approved by the Northwestern University IRB. All patients provided informed consent. All animal studies were reviewed and approved by the Institutional Animal Care and Use Committee at Northwestern University (PHS assurance no. A328301).

### Data availability.

All original files for RNA-Seq data were submitted to NCBI’s Gene Expression Omnibus database (bulk RNA-Seq data, GSE221781; single-cell RNA-Seq data GSE221987).

## Author contributions

XR and RS conceived and designed experiments. XR, RS, LDM, JMU, and DPS conducted experiments. XR, RS, LDM, EBP, LKY, TMB, and CL analyzed data. XR, RS, HN, and ABH performed and analyzed multiplex immunofluorescence experiments on clinical specimens. RS, PSD, EBT, and ABH contributed reagents, materials, or analysis tools. XR and RS wrote the manuscript. LDM, EBP, DPS, LKY, TMB, PSD, HN, CL, EBT, and ABH edited the manuscript.

## Supplementary Material

Supplemental data

Supplemental video 1

Supplemental video 2

Supplemental video 3

Supplemental video 4

Supporting data values

## Version 1 06/01/2023

In-Press Preview

## Version 2 08/01/2023

Electronic publication
