# Supplementary material for: Macrophage–endothelial cell crosstalk orchestrates neutrophil recruitment in inflamed mucosa
Source: J Clin Invest. 2023 Aug 1;133(15):e170733. doi: 10.1172/JCI170733 (PMC10378177; doi:10.1172/JCI170733)
Supplement: Supplemental data [file jci-133-170733-s092.pdf]

Macrophage-Endothelial Cell Crosstalk Orchestrates Neutrophil Recruitment in Inflamed Mucosa

Authors: Xingsheng Ren[1], Laura D. Manzanares[1], Enzo B. Piccolo[1], Jessica M. Urbanczyk[1], David P. Sullivan[1], Lenore K. Yalom[1], Triet M. Bui[1], Connor Lantz[1], Hinda Najem[2], Parambir S. Dulai[3], Amy B. Heimberger[2], Edward B. Thorp[1], Ronen Sumagin[1]

Short title: Vascular macrophages regulate neutrophil migration

Affiliations: [1]Department of Pathology, Northwestern University Feinberg School of Medicine, 300 East Superior St. Chicago, IL 60611. [2]Department of Neurological Surgery and Malnati Brain Tumor Institute of the Lurie Comprehensive Cancer Center, Feinberg School of Medicine, Northwestern University, Chicago, Illinois, USA. [3]Department of Medicine, Gastroenterology and Hepatology, Northwestern Memorial Hospital, 259 E Erie St, IL 60611.

Correspondence: Ronen Sumagin, PhD. Department of Pathology, Northwestern University Feinberg School of Medicine, 300 East Superior Street, Chicago, IL 60611. E-mail: ronen.sumagin@northwestern.edu

Key words: Inflammation, adhesion molecules, migration, recruitment, ICAM-1, mucosa

Disclosures: The authors have declared that no conflict of interest exists.

Materials and Methods

Animals: C57BL6J and TNFα knockout (KO) mice (B6.129S-Tnftm1Gkl/J) were purchased from Jackson Laboratories (Bar Harbor, ME). CX3CR1-enhanced green fluorescence protein (EGFP) reporter mice were a gift from Dr. Harris Perlman (Northwestern University, NU, Chicago, IL). All animals were maintained under specific pathogen-free conditions at the Northwestern University Feinberg School of Medicine animal facilities. At the end of all experimental procedures, animals were euthanized via cervical dislocation. All experimental protocols were approved by the Institutional Animal Care and Use Committee.

Cells: Mouse microvascular brain endothelial cell (bEnd-3) were grown in Dulbecco's modified Eagle's medium (DMEM) supplemented with 10% fetal bovine serum (FBS), 1% L-glutamine, and 1% penicillin-streptomycin. Human umbilical endothelial cells (HUVECs) were grown in human endothelial serum-free media supplemented with 1% L-glutamine, endothelial cell growth supplement (0.0375 mg/mL), 10% FBS, 1% penicillin-streptomycin, and 1% non-essential amino acids. Human PMNs were isolated from blood obtained from healthy volunteers by density gradient centrifugation (1, 2) and handled according to protocols for the protection of human subjects, as approved by the Northwestern University Institutional Review Board. PMNs were used for experiments within 2 hours of isolation. Murine PMNs were isolated from bone marrow (BM) and enriched to ~90% purity using Histopaque gradients (1077 and 1119, Sigma) as previously described (3, 4). The human monocytic cell line THP-1 was obtained from the ATCC (Manassas, VA) and maintained in RPMI 1640 medium, supplemented with 10% fetal calf serum, 1% L-glutamine, and 0.05 mmol/L 2-mercaptoethanol. THP-1 cells were differentiated using phorbol 12-myristate 13-acetate (PMA; 100 nmol/L) for 5 days before use. Mouse BM-derived macrophages were isolated as previously described(5). Briefly, BM cells were flushed from the

femur and tibia with serum-free Dulbecco modified Eagle's medium and red blood cells were lysed by sequential treatment with 0.02% and 1.6% NaCl. Cells were plated at 6×10^6 cells per 10cm non-treated culture dish and differentiated into macrophages by adding 1×10^4 U/mL macrophage colony-stimulating factor (MCSF) into the growth media. On day 6, macrophages were stimulated with LPS/interferon-γ (IFNγ) (50 and 20 ng/mL, respectively) for 24 hours for acquisition of inflammatory phenotype.

Reagents and Antibodies: RPMI 1640 and Dulbecco modified Eagle's medium growth media, L-glutamine, penicillin, streptomycin, and nonessential amino acids were obtained from CellGro (Manassas, VA). FBS was obtained from Atlanta Biologicals (Atlanta, GA). N-formyl-l-methionyl-leucyl-l-phenylalanine, PMA, Hanks balanced salt solution with Ca^{2+} and Mg^{2+} (Hank balanced salt solution), LPS (E. Coli derived, O111:B4) and tumor necrosis factor α (TNFα) were from Sigma-Aldrich. Human/murine IFNγ were from PeproTech (Rocky Hill, NJ). Macrophage colony-stimulating factor was from eBioscience (San Diego, CA). Pacific Blue™ anti-mouse CD45 (clone 30-F11), PE and APC anti-mouse CD54 (clone YN1/1.7.4), APC anti-mouse TNFRI, PE (clone 55R-286), anti-mouse TNFRII (clone TR75-89), PE/Cyanine7 anti-mouse CD64 (clone X54-5/7.1) and APC/Cyanine7 anti-mouse/human CD11b (clone M1/70) were from BioLegend (San Diego, CA). The rat IgG2b isotype (clone LTF-2), anti-mouse TNFR2 (clone TR75-54.7), TNFα (clone XT3.11) and CSF1R (clone AFS98) antibodies were from BioXCell (Lebanon, NH). Anti-mouse PECAM-1 (clone 390) was purchased from Merck KGaA (Darmstadt, Germany) and labeled with DyLight 650 Fluorochrome purchased from Thermo Fisher Scientific (Waltham, MA). PE anti-mouse Ly6G (clone 1A8) and PE anti-mouse F4/80 (clone BM8) were from BD Biosciences (San Jose, CA, USA). PE anti-mouse ICAM-2 (clone 3C4) antibody and

CellTracker™ Orange CMTMR Dye were from Invitrogen (Waltham, MA). PerCP anti-mouse LYVE-1 antibody was purchased from Novus Biologicals (Centennial, CO).

Gut Intravital Imaging: Gut imaging has been performed as previously described (6). Briefly, control or LPS-treated mice (to induce gut inflammation) were anesthetized and a small vertical incision was made to expose a segment of the small intestine (near ilium). Mice were placed on a heated platform (37°C) and the exposed intestinal segment was secured onto a custom-made chamber with minimal tissue manipulation. To visualize blood vessels a non-blocking anti-PECAM-1 Ab (clone 390) conjugated to DyLight®−647 was administered i.v. (retroorbital) 30 minutes prior to imaging. Imaging was performed using an Olympus BX-51WI Fixed-Stage illuminator equipped with a Yokogawa CSU-X1-A1 spinning disk, a Hamamatsu Electron Multiplying Charge Coupled Device C9100-50 camera and a Modular Laser System with solid-state diode lasers with diode-pumped solid-state modules for 488, 561, and 640 nm and the appropriate filters (all assembled by Perkin Elmer, Naperville, IL). Z-axis movement and objective positioning were controlled by the Piezoelectric MIPOS100 System (Piezoystem, Jena, Germany). Images were collected using a 20X water-immersion objective (numeric aperture, 1.00). Volocity software (version 6.3; Perkin Elmer, Waltham, MA) was used to drive the microscopy and for image acquisition, which then were analyzed using ImageJ software version 1.8.0 (NIH, Bethesda, MD; https://imagej.nih.gov/ij).

In situ labeling and quantification of EC adhesion molecules. To measure changes in ICAM-1/2 expression, mucosal vessels were perfused with primary labeled anti-ICAM-1/2 Abs combined with anti-PECAM-1 Ab as above for plane normalization (7) (retroorbital injection). Whole mounts were prepared for imaging by excising intestinal segments and immobilizing them on custom made silicon plates. Following image acquisition, fluorescence intensity levels, as an index

of ICAM-1/2 expression levels, were analyzed by projecting a line 3 pixels wide along the wall at the central plane of the vessel cross section and obtaining the intensity profile along that line. The overall fluorescence was quantified as relative intensity using the intensity of PECAM-1 staining. For ICAM-1 hot spots quantification, vessels were partitioned into 25µm vessel segments and regions with intensity of at least 2-fold or greater than the relative overall expression of a 100µm segment were counted. For all intensity measurements, the laser power and camera gain settings were held constant.

Analyses of PMN-EC interactions: For analyses of cell fluxes, PMN rolling and adhesion, 30 seconds recording of random fields containing a 30- to 50µm postcapillary venule with steady flow were made using appropriate fluorescence illumination channels at an acquisition rate of 10 frames per second. Rolling cells were defined as cells that have remained in continuous contact with the vessel wall for greater than 5 seconds. Adherent cells were defined as cells that remained attached to the vessel wall for >20 seconds. Transmigrated/tissue PMNs were counted per field of view and VAMs were quantified along 100µm segments of the vessel wall.

Bone Marrow Transfer and Generation of Chimeric Mice: Bone marrow chimeras were generated and maintained using a standard protocol as previously described (8, 9). Briefly, ten-week-old CX3CR1-EGFP (heterozygous) reporter mice were lethally irradiated with a 1000-cGy dose using a Gammacell 40 Exactor 137Cs irradiator (Best Theratronics, Ottawa, ON, Canada) and reconstituted with bone marrow from 8-week-old C57BL/6 WT and TNF KO mice 24 hours after whole-body irradiation. Bone marrow donors and recipients were sex matched. Recipients were allowed to recover for up to 8 weeks and were given trimethoprim and sulfamethoxazole antibiotics in drinking water to prevent opportunistic infections. Reconstitution was confirmed by

fluorescence imaging of intestines for the presence of CD45+ immune cells and F4/80+ intestinal macrophages as well as by the absence of host-EGFP positive tissue resident cells.

Quantibody cytokine array: Mouse BM-derived macrophages were differentiated and stimulated with LPS/IFNγ. Following stimulation (day 6), cells were washed to remove LPS/IFNγ and 24 hours later, cell supernatants were collected and cleared of cellular debris by centrifugation (5 minutes at 1500 RPM followed by 10 minuntes at 13000 RPM). Macrophage supernatants were analyzed with a targeted 40-target chemokine array (Mouse Cytokine Array Q5, #QAM-CYT-5-1, RayBiotech, Atlanta, GA). Protein expression data from four technical replicates were averaged and normalized to background readout using Gene Cluster 3.0 and Java TreeView.

Flow Cytometry: Single cell suspensions following the relevant treatment were analyzed using a BD LSR Fortessa X-20 (Becton Dickinson, Franklin Lakes, NJ) instrument and FlowJo 10.7 software (Becton Dickinson) as previously described (10).

Multiplex immunofluorescence (IF) staining: The multiplex panel included the following unconjugated antibodies: CD31 (endothelial cells; Abcam EPR3131), CD66b (neutrophils; Novus Biologicals G10F5) and CD68 (pan monocyte/macrophage; Dako Agilent PG-M1). All antibodies were validated using conventional immunohistochemistry and/or IF staining, in conjunction with the corresponding fluorophore and the spectral 4',6-diamidino-2-pheynlindole (DAPI; ThermoFisher Scientific) counterstain. For optimal concentration and best signal/noise ratio all antibodies were tested at three different dilutions, starting with the manufacturer-recommended dilution (MRD), then MRD/2 and MRD/4. Secondary Alexa fluorophore 555 (ThermoFisher Scientific) and Alexa fluorophore 647 (ThermoFisher Scientific) were used at 1/200 and 1/400 dilutions respectively. All runs of the multiplex panel were executed using sequential IF methodology integrated in the Lunaphore COMET™ platform. Staining was performed using

automated cycles of two antibodies staining at a time, followed by imaging and elution, where no sample manipulation is required. Images stacking and visualization were done immediately after concluding the staining procedure. For image analyses, slides were automatically scanned using the Lunaphore COMET™ with fluorescent high-power field scan (20X magnification microscope). Fluorescent signals (DAPI, TRITC and Cy5) were captured separately at the corresponding fluorophore wavelength and then stacked in one image (OME.TIFF) without disrupting the unique fluorescent spectral signature of the markers. At least N=8 regions per sample were captured and quantification of the different populations of interest was conducted using ImageJ software.

Bulk RNA sequencing of ECs: ECs were FACS-sorted from digested, LPS-stimulated colon mucosa with and without macrophage depletion by gating on CD45-/Live1-/CD31+ cells. Total mRNA was extracted from sorted ECs using RNeasy Plus Mini Kit (Qiagen), followed by quantification with Qubit and RIN quality assessment by 2100 Agilent Bioanalyzer (Agilent Technologies, Santa Clara. CA). Low-input total cDNA library construction and Illumina sequencing: RNA-seq was conducted at the NUSeq Core Facility using a Illumina HiSeq 4000 NGS System (Illumina, San Diego, CA) to generate 50 bp reads with 300 million paired-end reads and 25 million reads per sample.

RNA sequencing data analyses: Raw reads were de-multiplexed into FASTQ format using bcl2fastq software (Illumina). The FASTQ files were quality checked for called bases of each read. Pre-processing of raw data was performed by Trimmomatic option in Chipster followed by removal of the Illumina adapters using the cutadapt program. Trimmed reads were aligned to the Mus musculus genome mm10 using STAR RNA-seq aligner (version STAR_2.5.1). Read counts for individual genes were generated using the HTseq framework (v.0.6.1p1). Differentially

expressed genes (DEGs) between macrophage-intact and macrophage-depleted conditions were determined following normalization for sequencing depth, variance-stabilizing transformation DESeq2 (v1.16.1), employing the Wald test to shrink Log-Fold-Changes (LFC) and the standard errors. The significance cut-off for DEGs was set at FDR-adjusted $p<0.05$ and $|log2FC| <1$ using the Benjamini-Hochberg method. All genes with normalized counts of <100 were removed from the data set and not considered for future analysis. A heatmap of variable genes of interest was created using gplots package.

Gene Ontology (GO) enrichment pathway analysis were performed using Metascape with Clarivate Analytics [http://metascape.org] with DEGs identified by DESeq2 as inputs 179. Outputs identified functionally related genes within characterized GO terms as well as coordinated changes in pathways significantly overrepresented within the datasets. Several sets of genes featuring ECs activation, including adhesion and junctional molecules, chemokines, caspases, and integrins were manually curated from previously published datasets.

Clinical specimens and single cell RNA sequencing analyses: Colonic biopsies were obtained from consented ulcerative colitis (UC) patients with Mayo endoscopic scores of 2-3 during routine screening. All procedures including biopsy collections and tissue processing were approved by the Northwestern University Institutional Review Board protocols (IRB). Biopsies were collected into DMEM media tubes, immediately transported to the lab, minced and digested by Liberase TH (0.1WU/mL Sigma #5401135001) and DNase I (0.5U/mL, Roche #10104159001) in HBSS using 3 rounds of 10 minute incubations (30 minutes total) at 37°C at 800RPM. The resulting cell suspensions were washed and passed through a 40μm strainer to ensure single cell separation. Following digest, cell viability was determined by Cellometer Auto 2000 Cell Viability Counter

(Nexcelom Bioscience) at the NUseq Core. Prior to single-cell RNA sequencing, cell concentration was adjusted to 400 cells/μl and standard cell viability threshold of >70%.

Single cell library generation: Libraries were generated using 10X Genomics Single Cell 3' Reagents Kit (v3.1) following manufacturer's protocols. Single cell suspensions were diluted in PBS with 1% BSA to target a recovery of approximately 10,000 cells per sample. Single cells were sequenced to a read depth of ~30,000 reads per cell. The single cell libraries were sequenced on the Illumina NovaSeq 6000 in collaboration with the Northwestern University Sequencing Core.

Data analyses: Cell Ranger (v6.0) was employed to demultiplex the raw base-call files into FASTQ files. Next, FASTQ files were aligned to the GRCh38 human reference genome, filtered, barcode counted, and unique molecular identifier (UMI) counted to generate feature barcode matrices for downstream analyses using Seurat (v3.1.0). To exclude low-quality data, cell barcodes with less than 300 UMIs, greater than 20% mitochondrial genes or greater than 45% ribosomal genes were filtered out from each sample. Next, SCTransform v2 was used for normalization and variance stabilization of the data using regularized negative binomial regression. Batch effects between each sample were minimized using the *integration* command in Seurat. To reduce the dimensionality of the expression matrices, a principal component analysis (PCA) was performed. The top ten principal components were utilized for dimensionality reduction and visualization of the data. Clusters were identified using graph-based clustering with resolutions between 0.1 - 0.3. Clusters were initially annotated based on Human Primary Cell Atlas database (HPCA, *celldex* package). These annotations were corroborated with common lineages markers to determine specific cell subtypes for downstream analyses, specifically PMNs, cluster 0: *G0S2, S100A8, HCAR3, CSF3R, S100A9, PROK2*; ECs, clusters 14 and 24: *PECAM1, PLVAP, VWF, SELE* and macrophages, cluster 7 and 9: *CD68, LYZ, CSF1R, CD163, MS4A7*. Differentially expressed genes

(DEGs) for each cell cluster were identified by Wilcoxon rank sum test with a threshold of 0.25 for the log fold-change.

Cell-Cell communication: CellChat v1.6.0 was used to examine potential cell-cell interactions between the annotated cell clusters within IBD patient biopsies. Briefly, we inferred biologically significant cell-cell communication by integrating gene expression with prior known knowledge of signaling ligands, receptors, and their cofactors. To determine communication probabilities at the pathway level, communication probabilities of all ligand-receptor interactions associated with each signaling pathway were summarized. To identify altered signaling sources and targets, we performed network centrality analysis computing the outdegree as measurement of likelihood of being signaling sources and indegree being likelihood of being signaling targets. The outdegree and indegree centralities are computed by summarizing each cell population-associated outgoing and ingoing communication probabilities across all signaling pathways. Signaling pathways of interest (specifically TNFα, IL1, and CCL signaling pathways) were visualized using a circle plot to view the dominant senders and receivers of each type of signaling.

Data availability: All original files for RNA-Seq data was submitted to NCBI's Gene Expression Omnibus database with the following accession numbers: GSE221781 - bulk RNA sequencing data and GSE221987 - single cell RNA sequencing Data.

Statistics: Statistical significance was assessed by 2-tailed student t-test or by one-way ANOVA with Newman-Keuls Multiple Comparison Test using Graphpad Prism (V4.0) for normally distributed data evaluated using the Shapiro Wilk test. Statistical significance was set at $p<0.05$. For all experiments data are shown as ± standard error of means (SEM).

Supplementary Figures

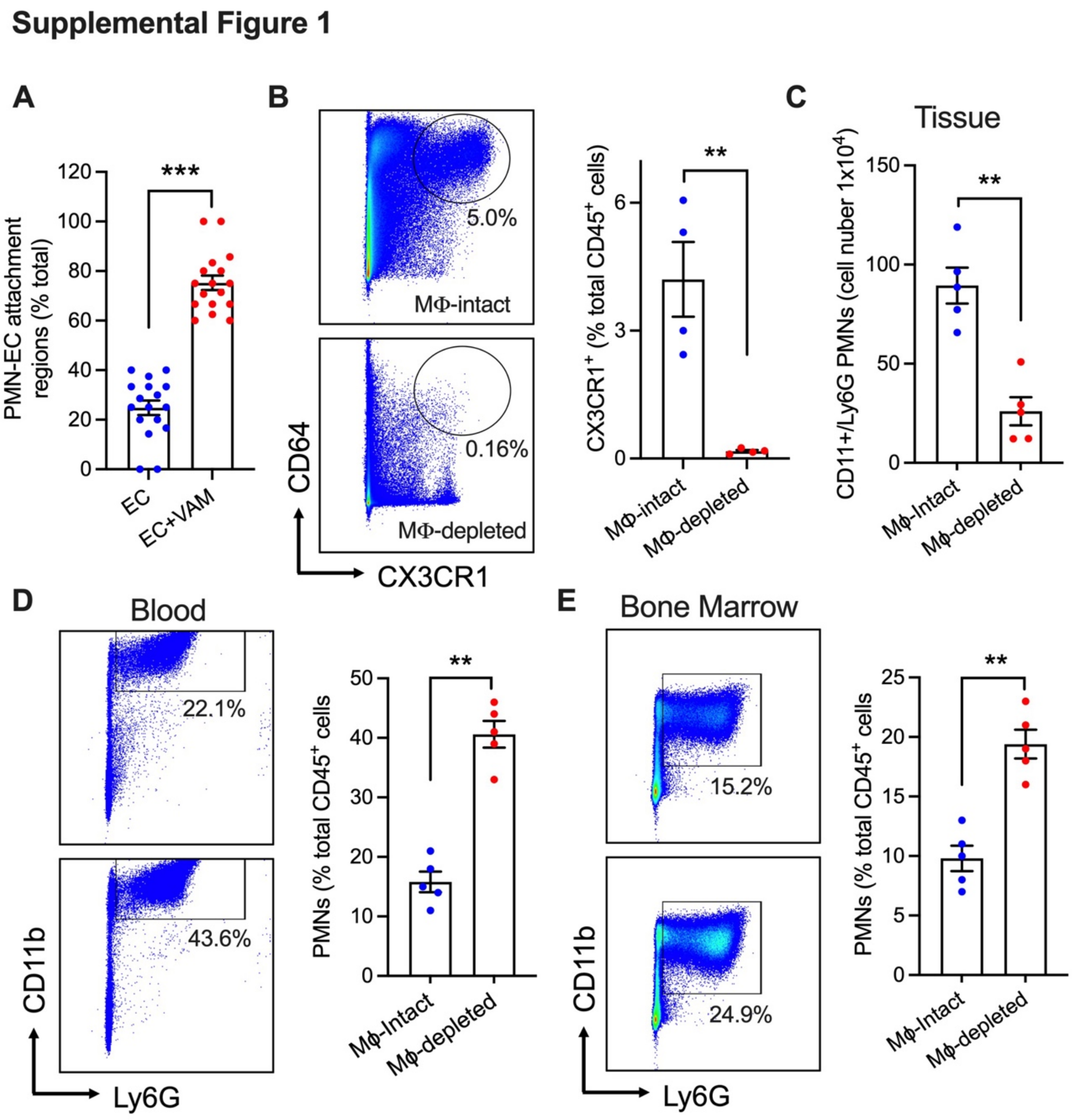

Supplemental figure 1: Macrophage depletion impacts PMN trafficking in the blood and the bone marrow. (A) PMN attachment (2 or more) to ECs in contact (EC+VAM) or not in contact (EC) with VAMs was quantified from whole mount confocal microscopy in LPS stimulated CX3CR1-EGFP macrophage that were in situ co-stained with anti-Ly6G antibody to mark

circulating PMNs. **(B)** Representative flow diagram and quantification of macrophage depletion in gut lamina propria using CSF-1R Ab (400mg/mouse, every other day for 3 weeks). Data are shown as percent CX3CR1+/CD64+ macrophage out of total $CD45^{+}$ immune cells. **(C)** Quantification of the PMN numbers in gut lamina propria. Data are shown as absolute numbers. **(D-E)** Representative flow diagrams and quantification of PMN fraction (percent Ly6G+/CD11b+ cells out of a total $CD45^{+}$ immune cells) in **(D)** blood and **(E)** bone marrow with/without macrophage depletion. N=4-5 independent experiments per condition. **$p < 0.01$. Two-sided student's t test, data represent mean ± SEM.

Supplemental Figure 2

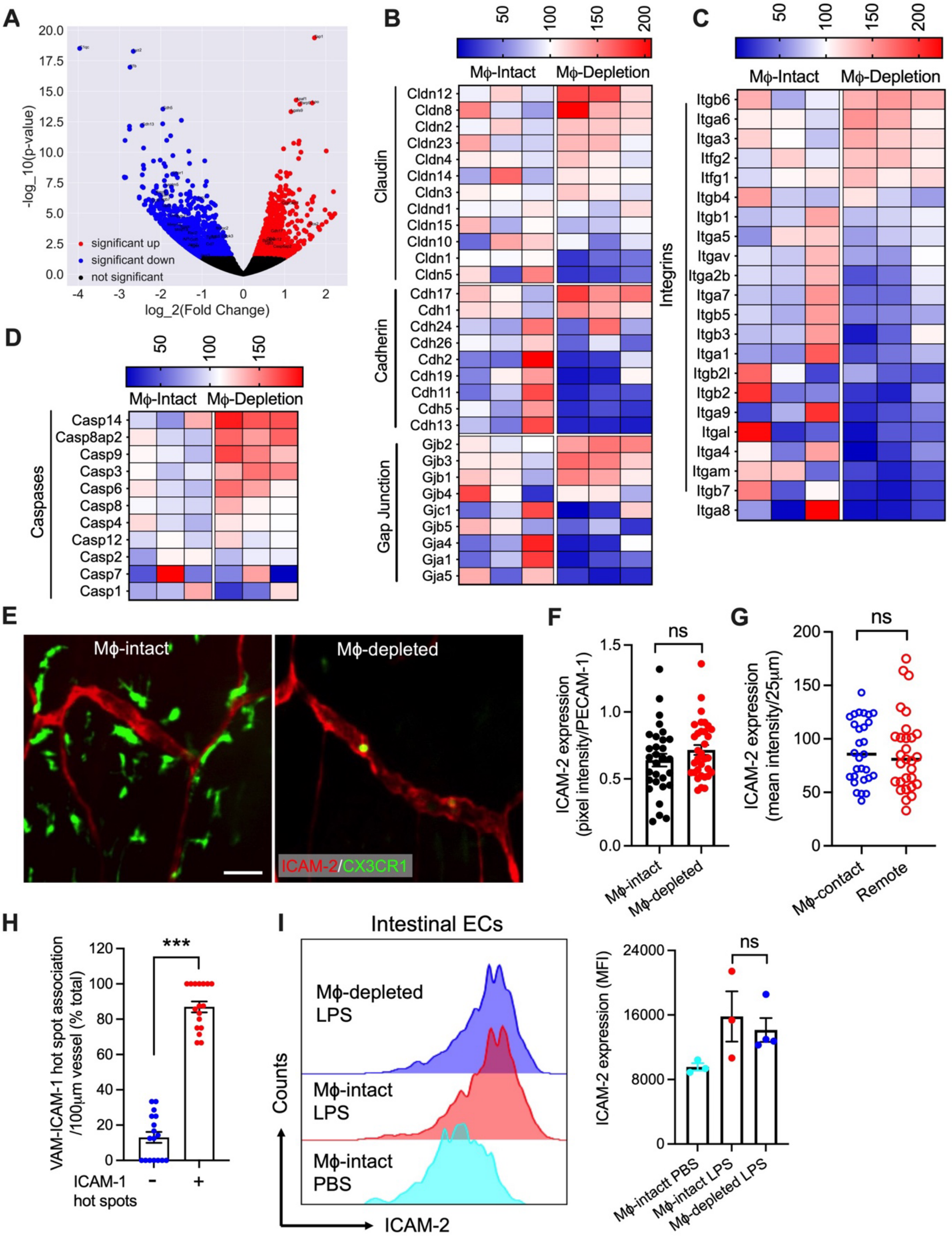

Supplemental figure 2: Macrophage depletion impacts EC function, but not ICAM-2 expression. (A-D) CD45-/Live1-/CD31+ ECs were flow-sorted from LPS-stimulated intestinal lamina propria with and without macrophage depletion and subjected to mRNA sequencing. **(A)** Volcano plot shows DEGs distribution. “Red” indicates significantly upregulated and “blue” significantly downregulated transcripts. “Black” indicates not significant. **(B)** Heatmap representation of genes encoding junctional components, **(C)** integrins and **(D)** caspases in ECs. Color scale represents % change in gene expression. **(E-G)** In situ fluorescence labeling was performed for ICAM-2 with/without macrophage depletion. **(E)** Representative whole mount confocal microscopy images and **(F,G)** quantification of ECs ICAM-2 expression patterns. Scale bar 20μm. Intestinal macrophages and blood vessel were visualized by CX3CR1 (green) and ICAM-2 (red) staining respectively. **(H)** VAM association with EC ICAM-1 hot spots was quantified. Data are shown as % total VAMs with/without association with VAMs per 100μm vessel length. **(I)** Representative flow diagram and quantification of ECs ICAM-2 expression in gut LP. N=3 independent experiments per condition. ns-not significant. Two-sided student’s t test and one-way ANOVA with Tukey’s multiple comparison test. Data are presented as mean ± SEM.

Supplemental Figure 3

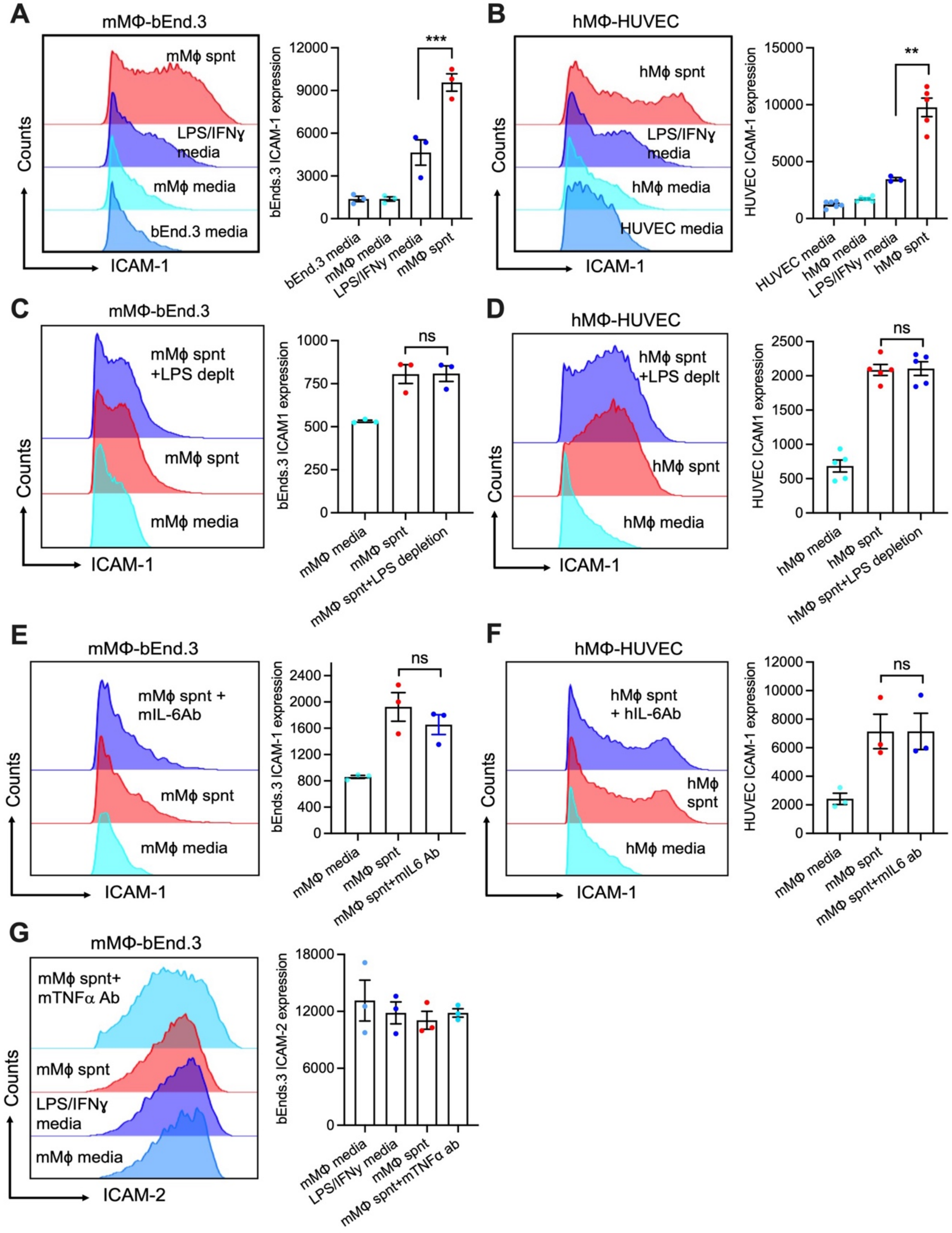

Supplemental figure 3: Impact of control and macrophage conditioned media on ECs ICAM-1 expression. (A,B) Representative flow diagrams and quantification of ICAM-1 expression in cultured mouse (bEnd.3) and human (HUVEC) ECs. LPS/IFNγ media was used as an experimental control for conditioned media from LPS/IFNγ treated macrophage. **(C,D)** Representative flow diagram and quantification of ICAM-1 expression in cultured bEnd.3 and HUVECs with/without the addition of anti-IL-6 inhibitory Abs. **(E)** Representative flow diagram and quantification of ICAM-2 expression in cultured bEnd.3 with/without the addition of anti-TNFα inhibitory Abs. N=3-4 independent experiments per condition. $^{**}p < 0.01$, $^{***}p < 0.001$. ns - not significant. One-way ANOVA with Tukey's multiple comparison test. Data are presented as mean ± SEM.

Supplemental Figure 4

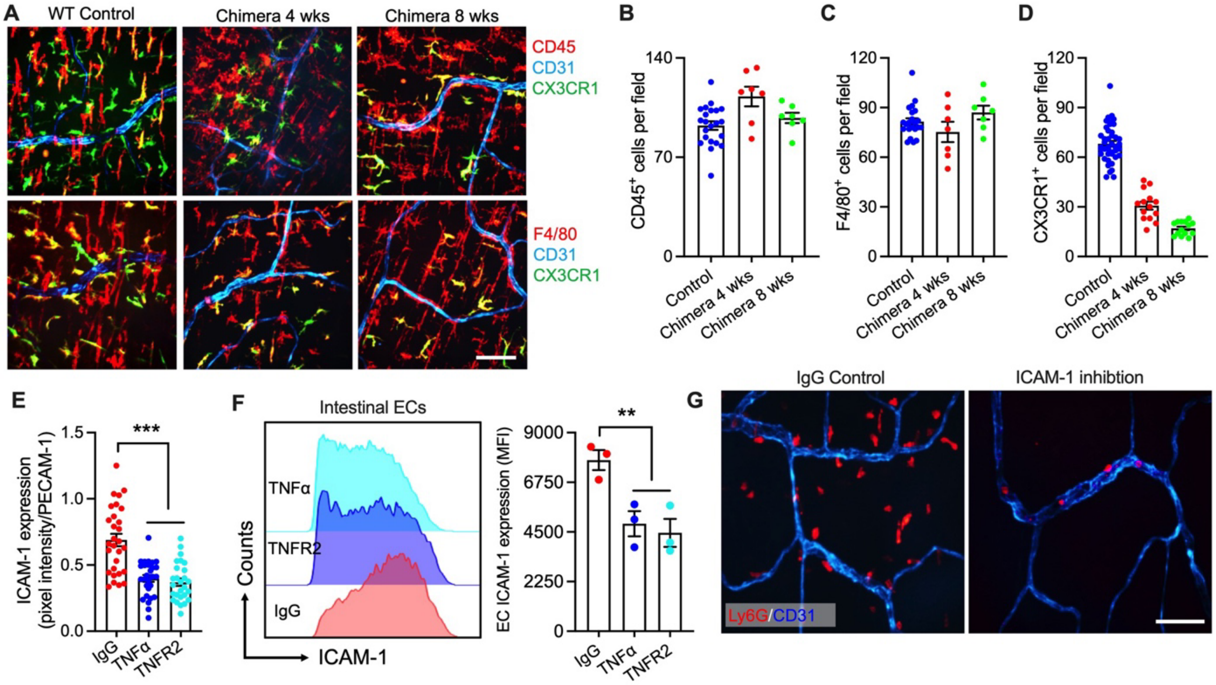

Supplemental figure 4: Intestinal tissue colonization by grafted macrophages in host CX3CR1-EGFP mice. Whole mount mucosal staining and confocal microscopy was performed on CX3CR-EGFP reporter mice at 4- and 8-weeks following grafting of WT, non-fluorescent donor cells. **(A)** Representative images used in quantification following staining for CD45 staining (red), upper panels and F4/80 (red) bottom panels. Host macrophages are CX3CR-EGFP (green). Vessels were outlined by CD31 staining (cyan blue). **(B-D)** Quantification of tissue CD45+, F4/80+ and the remaining host CX3CR1-EGFP+ cells overtime following bone marrow grafting. N=3 independent experiments per condition. **(E)** Animals were treated with control IgG or neutralizing Abs to TNFα or TNFR2 and *in situ* stained for ICAM-1. Fluorescence intensity as an index of ICAM-1 expression (red) relative to PECAM-1 (CD31, blue) was quantified. **(F)** Representative flow diagram and quantification depicting reduction in overall EC ICAM-1 expression following Ab-mediated inhibition of TNFα (400μg, ip.) or TNFR2 (400μg, iv.). (G)

Representative image of whole mount confocal microscopy depicting reduction in PMN tissue extravasation following Ab-mediated inhibition of ICAM-1 (400μg, iv.). Quantification shown in main Figure 6M. N=3-4 independent experiments per condition. ***$p < 0.001$. Two-sided student's t test and one-way ANOVA with Tukey's multiple comparison test. Data are presented as mean ± SEM.

Supplemental Figure 5

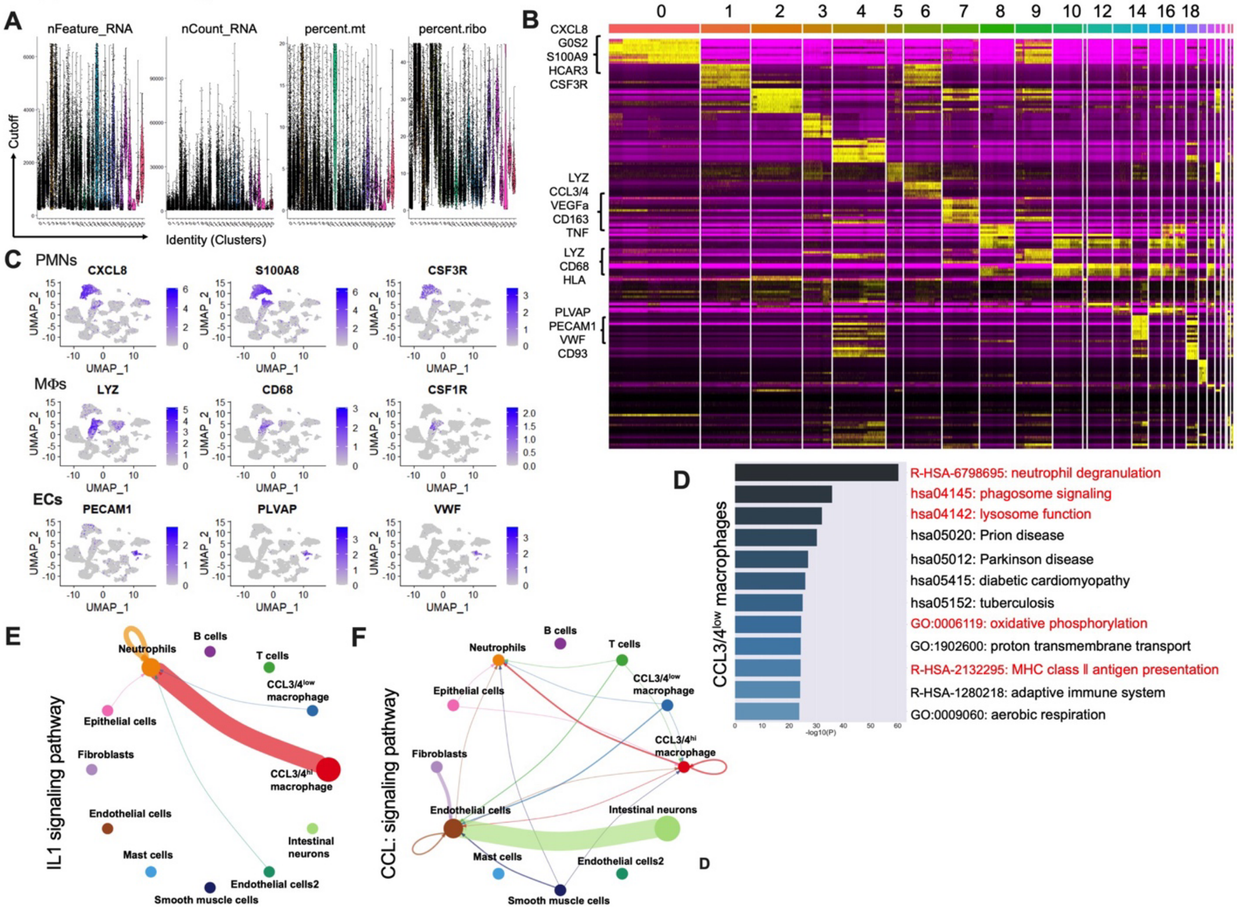

Supplemental figure 5: Single-cell sequencing analyses of human colon tissue from patients with IBD. (A) Quality control plots for single-cell RNA-Seq. **(B)** Unsupervised heatmap depicting relative gene expression (normalized and scaled z-scored) of cell clusters. Cluster 0 (PMNs), Cluster 7 (CCL3/4low macrophage), Cluster 9 (CCL3/4 high macrophage) and Cluster 14 (Endothelial cells) are defined based on their gene expressions (genes are shown on the left). **(C)** Plots feature expression of canonical and novel PMN, macrophage and EC subpopulations markers. **(D)** Enrichment analysis for Gene Ontology biological processes, pathways of Cluster 7 (CCL3/4low macrophage). **(E,F)** Outgoing communication patterns using CellChat analyses,

specifically focused on network centrality analysis of inferred IL-1 and CCL signaling pathway with macrophages defined as senders.

Supplemental Movies Legends

Supplemental Movie 1: Serosal imaging by IVM of the intestinal mucosa in CX3CR1-EGFP reporter mice following LPS-induced inflammation. Vessels are outlined by PECAM-1 fluorescence staining (blue) and PMNs by Ly6G staining (red). PMNs are seen to interact and firmly attach to the vessel wall in regions adjacent to VAMs. The time stamp indicates image acquisition in real time.

Supplemental Movie 2: Serosal imaging by IVM of the intestinal mucosa in CX3CR1-EGFP reporter mice following macrophage/VAM depletion by CSF-1R Ab and following LPS-induced inflammation. Vessels are outlined by PECAM-1 fluorescence staining (blue) and PMNs by Ly6G staining (red). Complete macrophage depletion is seen leading to abrogation of PMN adhesion. The time stamp indicates image acquisition in real time.

Supplemental Movie 3: Serosal imaging by IVM of the intestinal mucosa in LPS-stimulated BM-chimera mice, where WT marrow was grafted into irradiated WT reporter mice recipients (resulting in WT macrophages/VAMs). Vessels are outlined by PECAM-1 (blue) and PMNs by Ly6G (red) fluorescence staining. Robust PMN adhesion to ECs as in WT mice is seen. The time stamp indicates image acquisition in real time.

Supplemental Movie 4: Serosal imaging by IVM of the intestinal mucosa in LPS-stimulated BM-chimera mice, where TNFα-KO marrow was grafted into irradiated WT reporter mice recipients (resulting in TNFα-KO macrophages/VAMs). Vessels are outlined by PECAM-1 (blue) and PMNs by Ly6G (red) fluorescence staining. PMN adhesion to ECs is lost and only PMN rolling is seen. The time stamp indicates image acquisition in real time.

References

1. Butin-Israeli V, Houser MC, Feng M, Thorp EB, Nusrat A, Parkos CA, et al. Deposition of microparticles by neutrophils onto inflamed epithelium: a new mechanism to disrupt epithelial intercellular adhesions and promote transepithelial migration. FASEB J. 2016;30(12):4007-20.
2. Butin-Israeli V, Bui TM, Wiesolek HL, Mascarenhas L, Lee JJ, Mehl LC, et al. Neutrophil-induced genomic instability impedes resolution of inflammation and wound healing. J Clin Invest. 2019;129(2):712-26.
3. Slater TW, Finkielsztein A, Mascarenhas LA, Mehl LC, Butin-Israeli V, and Sumagin R. Neutrophil Microparticles Deliver Active Myeloperoxidase to Injured Mucosa To Inhibit Epithelial Wound Healing. J Immunol. 2017;198(7):2886-97.
4. Finkielsztein A, Mascarenhas L, Butin-Israeli V, and Sumagin R. Isolation and Characterization of Neutrophil-derived Microparticles for Functional Studies. J Vis Exp. 2018(133).
5. Amend SR, Valkenburg KC, and Pienta KJ. Murine Hind Limb Long Bone Dissection and Bone Marrow Isolation. J Vis Exp. 2016(110).
6. Sullivan DP, Bui T, Muller WA, Butin-Israeli V, and Sumagin R. In vivo imaging reveals unique neutrophil transendothelial migration patterns in inflamed intestines. Mucosal Immunol. 2018;11(6):1571-81.
7. Sumagin R, and Sarelius IH. TNF-alpha activation of arterioles and venules alters distribution and levels of ICAM-1 and affects leukocyte-endothelial cell interactions. Am J Physiol Heart Circ Physiol. 2006;291(5):H2116-25.
8. Sullivan DP, Dalal PJ, Jaulin F, Sacks DB, Kreitzer G, and Muller WA. Endothelial IQGAP1 regulates leukocyte transmigration by directing the LBRC to the site of diapedesis. J Exp Med. 2019;216(11):2582-601.
9. Duran-Struuck R, and Dysko RC. Principles of bone marrow transplantation (BMT): providing optimal veterinary and husbandry care to irradiated mice in BMT studies. J Am Assoc Lab Anim Sci. 2009;48(1):11-22.
10. Weber DA, Sumagin R, McCall IC, Leoni G, Neumann PA, Andargachew R, et al. Neutrophil-derived JAML inhibits repair of intestinal epithelial injury during acute inflammation. Mucosal Immunol. 2014;7(5):1221-32.
